# The Activation of p300 Enhances the Sensitivity of Pituitary Adenomas to Dopamine Agonist Treatment by Regulating the Transcription of DRD2

**DOI:** 10.3390/ijms252312483

**Published:** 2024-11-21

**Authors:** Sihan Li, Xingbo Li, Quanji Wang, Qian Jiang, Zihan Wang, Linpeng Xu, Yimin Huang, Ting Lei

**Affiliations:** 1 Sino-German Neuro-Oncology Molecular Laboratory, Department of Neurosurgery, Tongji Hospital of Tongji Medical College of Huazhong University of Science and Technology, Jiefang Avenue 1095, Wuhan 430030, China; lisihan@hust.edu.cn (S.L.); lxbhusttj@tjh.tjmu.edu.cn (X.L.); quanjiwang@hust.edu.cn (Q.W.); m202076460@hust.edu.cn (Q.J.); wzhneuros@hust.edu.cn (Z.W.); m202276296@hust.edu.cn (L.X.); 2 Hubei Key Laboratory of Neural Injury and Functional Reconstruction, Huazhong University of Science and Technology, Wuhan 430030, China

**Keywords:** prolactin-secreting pituitary adenoma, DA resistance, p300, histone acetylation, tanshinone IIA

## Abstract

Prolactinomas are commonly treated with dopamine receptor agonists (DAs), such as bromocriptine (BRC) and cabergoline (CAB). However, 10–30% of patients exhibit resistance to DA therapies. DA resistance is largely associated with reduced dopamine D2 receptor (DRD2) expression, potentially regulated by epigenetic modifications, though the underlying mechanisms are still unclear. Clinical samples were assessed for p300 expression. MMQ and AtT-20 cells were engineered to overexpress either wild-type p300 or a histone acetyltransferase (HAT) domain-mutant form of p300. Mechanistic studies included cell proliferation assays, flow cytometry, immunohistochemistry, immunofluorescence, co-immunoprecipitation, chromatin immunoprecipitation followed by quantitative PCR, reverse transcription quantitative PCR, and Western blotting. Additionally, an in vivo nude mouse xenograft model was used to confirm the in vitro findings. DAs downregulated p300 through the cAMP-PKA-CREB pathway. Activation of the HAT domain of p300 increased H3K18/27 acetylation, promoted DRD2 transcription, and worked synergistically with DA to exert anti-tumor effects both in vitro and in vivo. Tanshinone IIA (Tan IIA) upregulated p300 and DRD2, enhancing the therapeutic efficacy of BRC. These findings highlight the role of p300 in regulating DRD2 transcription in DA-resistant prolactinomas. Combining Tan IIA with BRC may offer a promising strategy to overcome DA resistance.

## 1. Introduction

Prolactinomas are the most common subtype of pituitary adenomas, accounting for approximately 40% of all pituitary tumors [1]. The combined effects of tumor mass and excessive prolactin secretion often lead to clinical symptoms such as headaches, visual impairment, amenorrhea, galactorrhea, and reduced sexual function. Dopamine agonists (DAs), such as bromocriptine (BRC) and cabergoline (CAB), are currently the first-line therapeutic agents for treating prolactinomas [2]. In the majority of patients, DAs effectively lower serum prolactin levels and reduce tumor size, thereby alleviating clinical symptoms. However, 20–30% of patients treated with BRC and 10% of those treated with CAB exhibit resistance to DAs, which is marked by insufficient tumor shrinkage, or inadequate prolactin level control [3]. Furthermore, these patients often experience tumor recurrence following surgical resection or radiotherapy [4], presenting substantial treatment challenges and adversely affecting their quality of life and prognosis.

Numerous studies have indicated that resistance to DA treatment in prolactinomas is largely due to diminished dopamine D2 receptors (DRD2) expression or reduced DA binding affinity [5]. The transcriptional regulation of DRD2 can be influenced by epigenetic modifications [6]. p300, a histone acetyltransferase, frequently forms a complex with CREB-binding protein (CBP) and regulates gene transcription through histone acetylation [7]. This complex plays a crucial role in processes such as cell proliferation, differentiation, apoptosis, and tumor drug resistance. In prolactinoma management, DAs target the DRD2, which are abundantly expressed on tumor cell membranes. This interaction activates the inhibitory Gα protein, subsequently suppressing the downstream cAMP/PKA/CREB signaling pathway, which reduces prolactin transcription and alleviates endocrine symptoms [8]. The CBP/p300 complex can be recruited by phosphorylated CREB to gene promoter regions, thereby regulating gene transcription [7]. In this study, we observed a significant reduction in p300 expression in DA-resistant prolactinoma tissues, suggesting that p300 may contribute to the mechanism of DA resistance. However, research on p300 and prolactinomas is limited, and few studies have investigated histone acetylation modifications as a mechanism underlying DA resistance.

In this study, we analyzed p300 expression levels in clinical samples of DA-resistant prolactinomas and confirmed the upstream regulatory mechanism of DA-mediated cAMP-PKA-CREB signaling. Using both in vivo and in vitro experiments, we demonstrated the synergistic anti-tumor effects of combining p300 activation with DA treatment and investigated the underlying mechanisms. Additionally, we identified Tanshinone IIA, a compound derived from traditional Chinese medicine, as an agent that upregulates p300 expression. This finding suggests a potential therapeutic strategy to enhance the effectiveness of DA treatment in DA-resistant prolactinomas.

## 2. Results

### 2.1. DA Downregulates p300 Expression in Pituitary Tumor Cells

**Figure 1:**
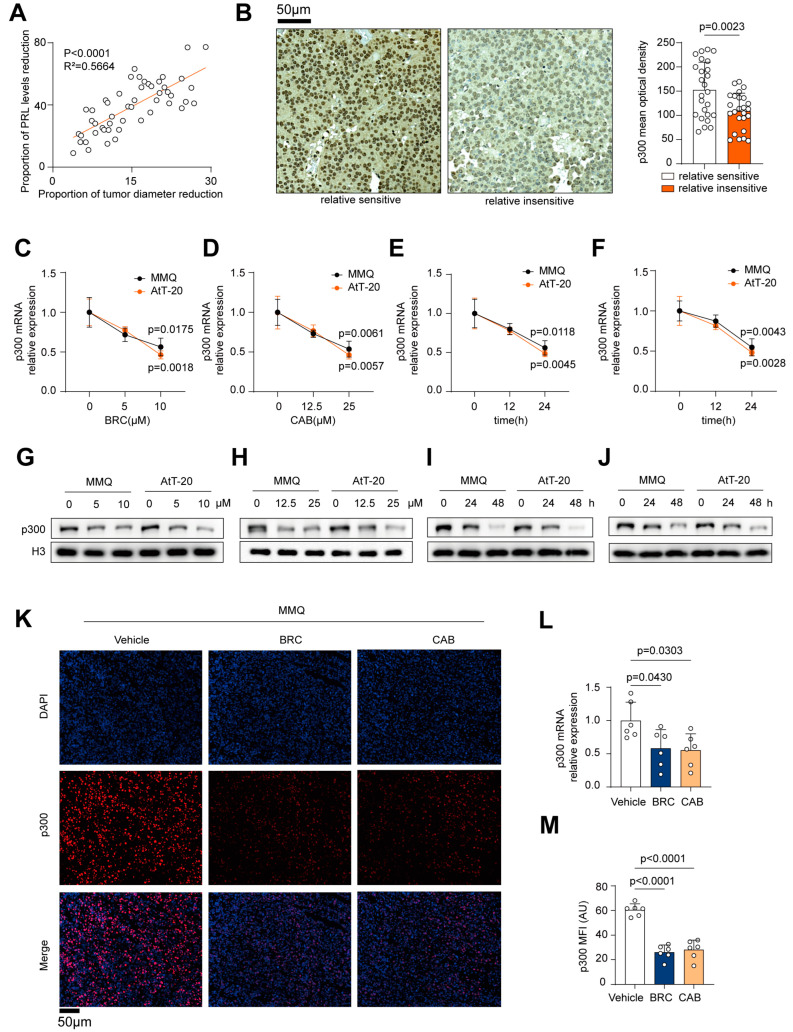
DA downregulates p300 expression in pituitary tumor cells. (**A**) A correlation analysis between the percentage reduction in the tumor maximum diameter and the percentage decrease in serum prolactin levels before and after BRC treatment in 50 patients with drug-resistant prolactinomas, classifying them into a relatively sensitive group (*n* = 25) and a relatively insensitive group (*n* = 25). (**B**) Immunohistochemistry (IHC) staining of p300 expression in the two patient groups, representative images (left), scale bar, 50 μm, quantification (right), *n* = 25. (**C**,**D**) q-PCR of p300 expression in MMQ and AtT-20 cells treated with BRC (0, 5, 10 μM) or CAB (0, 12.5, 25 μM) for 24 h, *n* = 3. (**E**,**F**) q-PCR of p300 expression in MMQ and AtT-20 cells treated with BRC (10 μM) or CAB (25 μM) for various periods (0, 12, 24 h), *n* = 3. (**G**,**H**) Western Blot (WB) analysis of p300 expression in MMQ and AtT-20 cells treated with BRC (0, 5, 10 μM) or CAB (0, 12.5, 25 μM) for 48 h, *n* = 3. (**I**,**J**) WB analysis of p300 expression in MMQ and AtT-20 cells treated with BRC (10 μM) or CAB (25 μM) for various periods (0, 24, 48 h), *n* = 3. (**K**–**M**) Subcutaneous tumor formation in nude mice using MMQ cells, followed by intraperitoneal injections of PBS, BRC (10 mg/kg/d), or CAB (15 mg/kg/d) for 2 weeks, q-PCR of p300 expression in MMQ (**L**) tumor tissues, immunofluorescence (IF) staining of Ki-67 expression in MMQ (**K**,**M**) tumor tissue sections, representative images (**K**), scale bar, 50 μm, quantification (**M**), *n* = 6. Data are shown as mean ± SD. Statistical analyses were conducted using Pearson’s correlation analysis, unpaired *t*-tests, and one-way ANOVA. Abbreviations: BRC, Bromocriptine; CAB, Cabergoline.

To investigate the association between p300 and drug resistance in patients, we collected data from 50 patients between January 2018 and January 2022 who exhibited resistance to BRC treatment and underwent surgery at the Department of Neurosurgery, Tongji Hospital. Based on the linear regression analysis of the reduction in tumor maximum diameter and serum prolactin levels before and after BRC treatment (Figure 1A), patients were divided into two groups using the median percentage of tumor reduction as a cutoff: 25 relatively sensitive and 25 relatively insensitive patients. Tumor tissues from both groups underwent q-PCR analysis and immunohistochemistry staining of p300 (Figure 1B, Supplementary Figure S1A). The results showed that relatively insensitive patients expressed lower transcriptional and protein levels of p300, suggesting that reduced p300 expression may be associated with tumor resistance. Supplementary Table S1 shows patients’ baseline characteristics and treatment efficacy.

Subsequently, MMQ and AtT-20 cells were cultured in vitro and treated with dopamine agonists (DA), including different concentrations of BRC and CAB, for varying periods (Figure 1C–J). We observed that both the transcriptional and protein levels of p300 were significantly reduced under DA treatment, indicating that DA can markedly downregulate p300 expression levels in MMQ and AtT-20 cells in vitro.

We further conducted an in vivo experiment with subcutaneous tumor xenografts in nude mice. After DA treatment, tumor tissues were subjected to q-PCR analysis and immunofluorescence staining of p300 (Figure 1K–M, Supplementary Figure S1B–D), revealing that DA also decreased p300 expression levels in the xenografted tumors.

These findings suggest that p300 is associated with drug resistance, and DA treatment can reduce p300 expression of MMQ and AtT-20 cells both in vitro and in vivo.

### 2.2. DA Downregulates p300 Expression Through Inhibition of the cAMP-PKA-CREB Pathway

**Figure 2:**
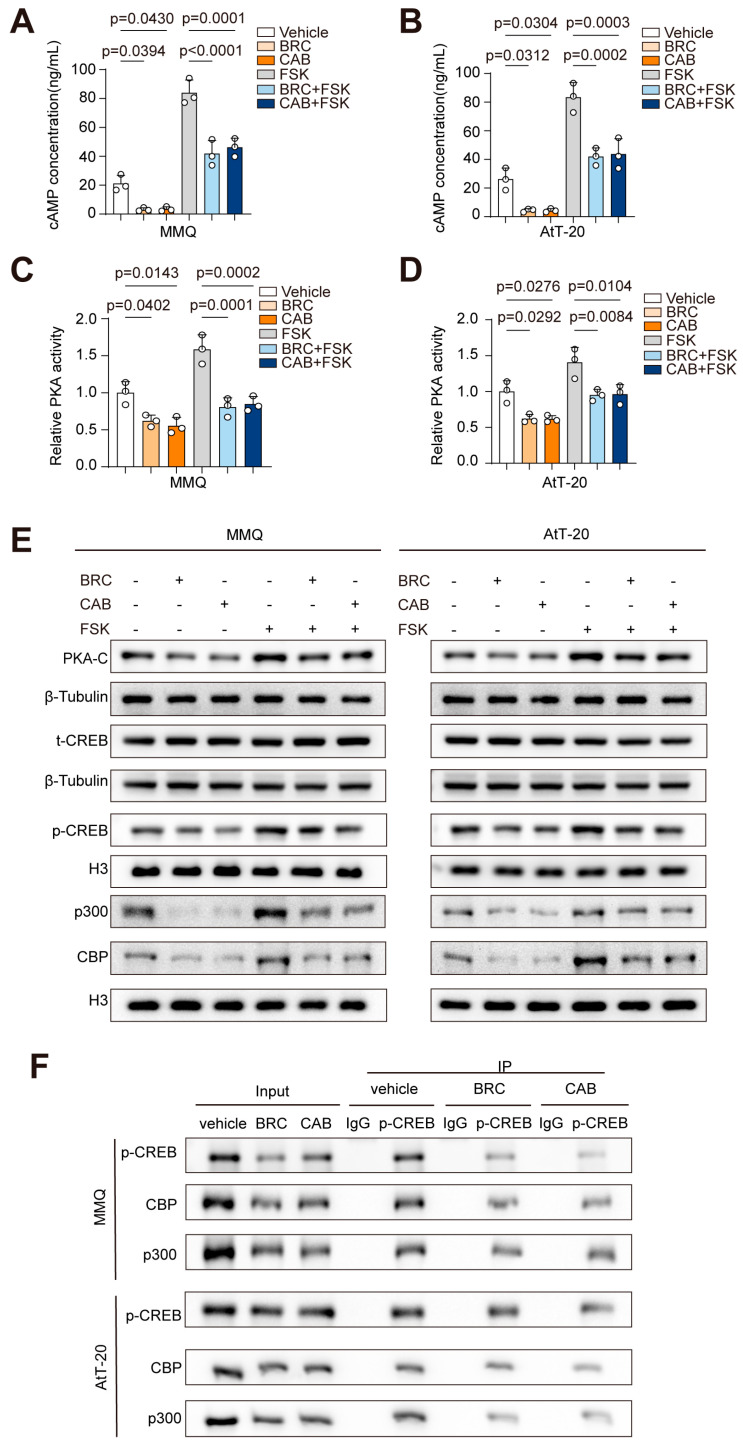
DA downregulates p300 expression through inhibition of the cAMP−PKA−CREB pathway. (**A**–**E**) MMQ and AtT-20 cells were treated with BRC (10 μM), CAB (25 μM), FSK (50 μM), BRC (10 μM) + FSK (50 μM), or CAB (25 μM) + FSK (50 μM) for 48 h; the cAMP ELISA assay of Intracellular cAMP concentrations in MMQ (**A**) and AtT-20 (**B**) cells, PKA activity assay of intracellular PKA activity in MMQ (**C**) and AtT-20 (**D**) cells, (**E**) Western Blot (WB) analysis of total PKA-C, total CREB (t-CREB), phosphorylated CREB (p-CREB), CBP, and p300 expression in MMQ and AtT-20 cells, *n* = 3. (**F**) Co-immunoprecipitation (Co-IP) experiments using p-CREB antibodies were performed in MMQ and AtT-20 cells treated with BRC (10 μM) and CAB (25 μM) for 48 h, followed by WB analysis of CBP and p300 expression, *n* = 3. Data are shown as mean ± SD. Statistical analyses were conducted using one-way ANOVA. Abbreviations: BRC, Bromocriptine; CAB, Cabergoline; FSK, Forskolin.

To further investigate the regulatory effect of DA on p300 expression, we analyzed the downstream cAMP/PKA/CREB signaling pathway following DA activation of the DRD2 receptor. Our findings demonstrated that DA significantly decreased intracellular cAMP concentrations in MMQ and AtT-20 cell lines (Figure 2A,B), accompanied by a reduction in PKA activity (Figure 2C,D). Moreover, the DA-induced reductions in cAMP levels and PKA activity were reversed upon forskolin-mediated elevation of intracellular cAMP.

Subsequently, Western blotting was performed to assess the levels of PKA-C and total CREB (t-CREB) in MMQ and AtT-20 cells under DA treatment, alone or in combination with forskolin. We also measured phosphorylated CREB (p-CREB), CREB-binding protein (CBP), and p300 levels in the nucleus (Figure 2E). The findings indicated that DA led to a reduction in PKA-C levels, along with a decrease in nuclear p-CREB, CBP, and p300 levels, effects that were reversible upon treatment with forskolin. Notably, t-CREB levels were unaffected. Additionally, immunoprecipitation assays further validated that CBP and p300 are capable of binding to p-CREB and are subject to regulation by DA (Figure 2F).

These results suggest that DA may mediate the downregulation of nuclear p300 expression by potentially inhibiting the cAMP-PKA-CREB pathway, thereby possibly diminishing the recruitment of p300 to the nucleus.

### 2.3. Activation of p300 HAT Activity Synergizes with DA to Exert Anti-Proliferative Effects in Pituitary Tumors Both In Vitro and In Vivo

**Figure 3:**
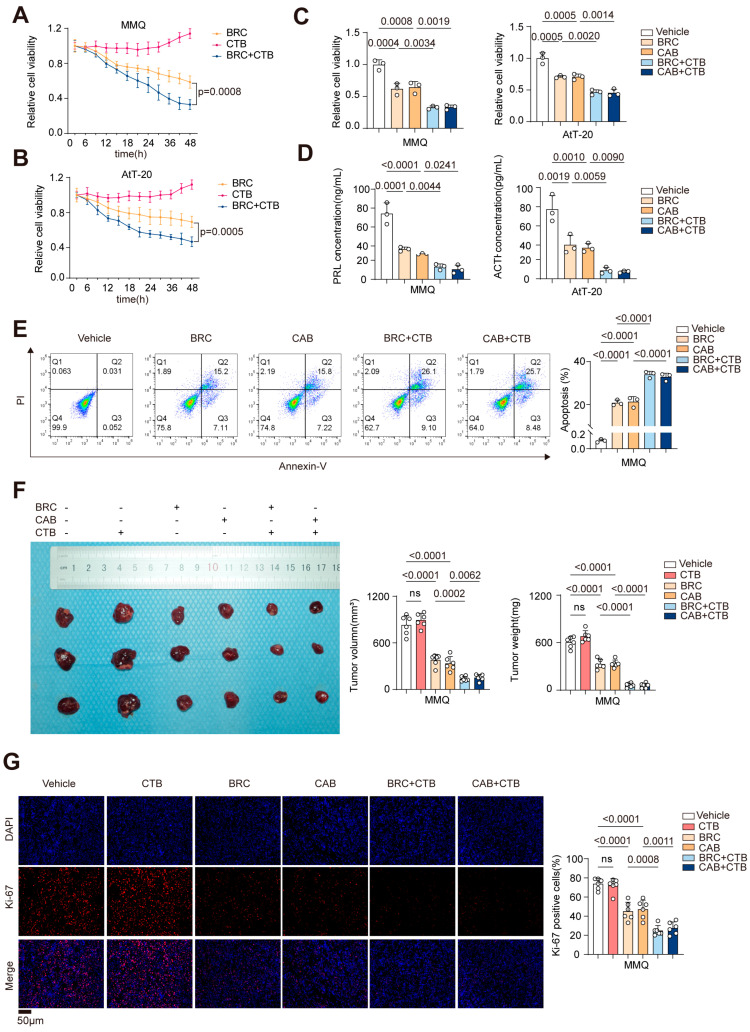
Activation of p300 HAT activity synergizes with DA to exert anti-proliferative effects in pituitary tumors both in vitro and in vivo. (**A**,**B**) CCK−8 assay of cell viability in MMQ (**A**) and AtT-20 (**B**) cells treated with BRC (10 μM), CTB (50 μM), or BRC (10 μM) + CTB (50 μM) for various periods (0, 3, 6, 9, 12, 15, 18, 21, 24, 30, 36, 42, 48 h), *n* = 3. (**C**–**E**) MMQ and AtT-20 cells were treated with BRC (10 μM), CAB (25 μM), CTB (50 μM), BRC (10 μM) + CTB (50 μM), or CAB (25 μM) + CTB (50 μM) for 48 h, (**C**) CCK-8 assay of cell viability, (**D**) PRL ELISA assay of supernatant prolactin concentration in the MMQ cells, and ACTH ELISA assay of supernatant ACTH concentration in the AtT-20 cells, (**E**) Annexin-V apoptosis flow cytometry analysis of cell apoptosis, quantification (right), *n* = 3. (**F**) Subcutaneous tumor formation in nude mice using MMQ cells, followed by intraperitoneal injections of PBS, BRC (10 mg/kg/d), CAB (15 mg/kg/d), CTB (20 mg/kg/d), BRC (10 mg/kg/d) + CTB (20 mg/kg/d), or CAB (15 mg/kg/d) + CTB (20 mg/kg/d) for 2 weeks, representative images of subcutaneous xenograft tumors (left), average volume of excised tumors (middle), average weight of excised tumors (right). (**G**) Immunofluorescence (IF) staining of Ki-67 expression in tumor tissue sections, scale bar, 50 μm, quantification (right), *n* = 6. Data were shown as mean ± SD. Statistical analyses were conducted using one-way ANOVA. Abbreviations: BRC, Bromocriptine; CAB, Cabergoline; CTB, N-(4-chloro-3-trifluoromethyl-phenyl)-2-ethoxy-benzamide); ns, not significant.

In order to examine the role of p300 expression levels in the context of DA treatment in MMQ and AtT-20 cells, we employed CTB (N-(4-chloro-3-trifluoromethyl-phenyl)-2-ethoxy-benzamide) to activate the p300 histone acetyltransferase (HAT) domain. This approach allowed us to observe its synergistic effect in conjunction with DA treatment in both cell lines. First, using CCK-8 assay, we evaluated the therapeutic effects of CTB alone, BRC alone, and their combination at various treatment times (0, 3, 6, 9, 12, 15, 18, 21, 24, 30, 36, 42, and 48 h) on both cell lines (Figure 3A,B). Furthermore, we validated the therapeutic efficacy of CAB combined with CTB on both cell lines (Figure 3C). The results showed that CTB alone promoted cell proliferation in both cell lines, consistent with previous reports. Interestingly, when combined with DA, a synergistic antitumor effect was observed.

Since MMQ and AtT-20 cells, as functional pituitary adenoma cell lines, secrete prolactin (PRL) and adrenocorticotropic hormone (ACTH) respectively, we performed ELISA assays (Figure 3D) and found that the combination therapy also exhibited a synergistic inhibitory effect on hormone secretion in both cell lines. Given that DA treatment of pituitary adenomas not only inhibits cell proliferation but also induces apoptosis, we used flow cytometry to assess apoptosis in the different treatment groups (Figure 3E, Supplementary Figure S2A). The results indicated that, compared to DA alone, the combination therapy significantly enhanced apoptosis in MMQ and AtT-20 cells.

In addition, we performed in vivo experiments on subcutaneous xenograft models of MMQ and AtT-20 cells, administering CTB and DA alone or in combination via intraperitoneal injections for two weeks. The combination therapy group showed significantly smaller tumor volumes and lighter tumor weights (Figure 3F, Supplementary Figure S2B). Ki-67 staining of the tumor sections further demonstrated a marked reduction in tumor proliferation in the combination therapy group (Figure 3G, Supplementary Figure S2C).

These findings suggest that compared to DA treatment alone, CTB combined with DA exhibits a synergistic antitumor effect both in vitro and in vivo. This indicates that activating the p300 HAT domain may enhance the therapeutic efficacy of DA in the management of drug-resistant prolactinomas.

### 2.4. p300 Promotes DRD2 Transcription by Increasing Histone H3K18/27 Acetylation

As a crucial histone acetyltransferase, p300 catalyzes the acetylation of specific histone lysine residues, thereby promoting transcriptional activity. Notably, the acetylation of H3K18 and H3K27 (H3K18/27ac) has been widely recognized to correlate with chromatin openness and gene activation. To explore the potential mechanism underlying the synergistic effect of activating the p300 HAT domain combined with DA treatment in MMQ and AtT-20 cells, we hypothesized that p300 might enhance the transcriptional level of DRD2 by regulating H3K18 and H3K27 acetylation, thus improving the efficacy of DA.

**Figure 4:**
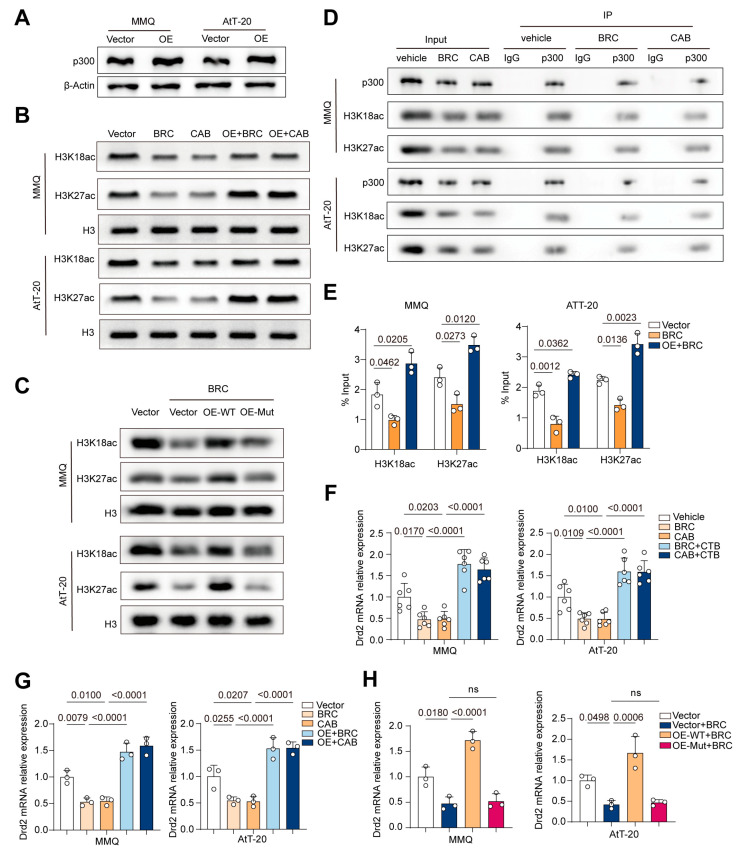
p300 promotes DRD2 transcription by increasing histone H3K18/27 acetylation. (**A**) Western Blot (WB) analysis of p300 expression in MMQ and AtT-20 cells with p300 overexpression, *n* = 3. (**B**) For MMQ and AtT-20 cells, both the vector group and the p300 overexpression (OE) group were treated with BRC (10 μM) and CAB (25 μM) for 48 h, WB analysis of H3K18ac and H3K27ac expression, *n* = 3. (**C**) For MMQ and AtT-20 cells, the vector group, p300 overexpression (OE) group, and p300 HAT mutant overexpression (OE-mut) group were treated with BRC (10 μM) and CAB (25 μM) for 48 h, WB analysis of H3K18ac and H3K27ac expression, *n* = 3. (**D**) Co-immunoprecipitation (Co-IP) experiments using p300 antibodies were performed in MMQ and AtT-20 cells treated with BRC (10 μM) and CAB (25 μM) for 48 h, followed by WB analysis of H3K18ac and H3K27ac expression, *n* = 3. (**E**) Chromatin Immunoprecipitation (ChIP) experiments using H3K18ac and H3K27ac antibodies were performed in MMQ and AtT-20 cells treated with BRC (10 μM) and CAB (25 μM) for 48 h, followed by qPCR detection of the enrichment levels of H3K18ac and H3K27ac at the DRD2 promoter region, *n* = 3. (**F**) q-PCR of DRD2 expression in MMQ and AtT-20 cells treated with BRC (10 μM), CAB (25 μM), CTB (50 μM), BRC (10 μM) + CTB (50 μM), and CAB (25 μM) + CTB (50 μM) for 48 h, *n* = 3. (**G**) For MMQ and AtT-20 cells, both the vector group and the p300 overexpression (OE) group were treated with BRC (10 μM) and CAB (25 μM) for 48 h, q-PCR of DRD2 expression, *n* = 3. (**H**) For MMQ and AtT-20 cells, the vector group, p300 overexpression (OE) group, and p300 HAT mutant overexpression (OE-mut) group were treated with BRC (10 μM) and CAB (25 μM) for 48 h, q-PCR of DRD2 expression, *n* = 3. Data were shown as mean ± SD. Statistical analyses were conducted using one-way ANOVA. Abbreviations: BRC, Bromocriptine; CAB, Cabergoline; CTB, N-(4-chloro-3-trifluoromethyl-phenyl)-2-ethoxy-benzamide); ns, not significant.

First, we established p300-overexpressing MMQ and AtT-20 cell lines (Figure 4A) and used Western blotting to assess the effect of DA on H3K18/27ac expression in these p300-overexpressing cells (Figure 4B). The results showed that DA reduced H3K18/27ac levels in both cell lines. Following p300 overexpression, compared to the vector group, the expression of H3K18ac was partially restored, while H3K27ac levels significantly increased.

Next, to confirm the importance of the p300 HAT domain, we constructed p300 mutants with defective HAT domains (Supplementary Figure S3B). Western blotting was used to examine changes in H3K18/27ac levels in cells overexpressing wild-type p300 (OE-WT) or mutant p300 (OE-Mut) after BRC treatment (Figure 4C). The results indicated that p300 HAT domain mutations negated the changes in H3K18/27ac levels caused by p300 overexpression, underscoring the critical role of HAT domain integrity. Subsequent immunoprecipitation experiments (Figure 4D) further demonstrated that H3K18/27ac could bind to p300 and be regulated by DA.

Since p300-mediated acetylation of H3K18 and H3K27 requires acetyl-CoA (Ac-CoA) as a substrate, we used ELISA to measure intracellular Ac-CoA levels at various times after BRC treatment (Supplementary Figure S3A). The results showed a significant reduction in Ac-CoA levels in MMQ and AtT-20 cells at 48 h, suggesting that decreased Ac-CoA may partially account for the DA-induced reduction in H3K18/27ac levels.

To explore the relationship between H3K18/27ac and DRD2 gene expression, we performed chromatin immunoprecipitation (ChIP) assays (Figure 4E). We found that BRC treatment significantly reduced the enrichment of H3K18/27ac in the DRD2 promoter region in both cell lines, while p300 overexpression markedly increased H3K18/27ac enrichment. This suggests that H3K18/27ac binds to the DRD2 promoter region, influencing chromatin accessibility.

Finally, qPCR was used to assess DRD2 transcription levels in MMQ and AtT-20 cells treated with DA alone, DA combined with CTB, or DA in cells overexpressing wild-type or mutant p300 (Figure 4F–H, Supplementary Figure S3C). The results showed that DA significantly reduced DRD2 expression levels in both cell lines, while the combination with CTB or p300 overexpression increased DRD2 transcription levels. In contrast, p300 mutants did not exhibit significant changes in DRD2 transcription compared to the BRC group.

These results suggest that p300 may regulate DRD2 transcription by modulating the levels of H3K18/27ac in the DRD2 promoter region.

### 2.5. Tanshinone IIA Upregulates p300 and Synergizes with BRC to Exert Anti-Tumor Effects in Pituitary Tumors

**Figure 5:**
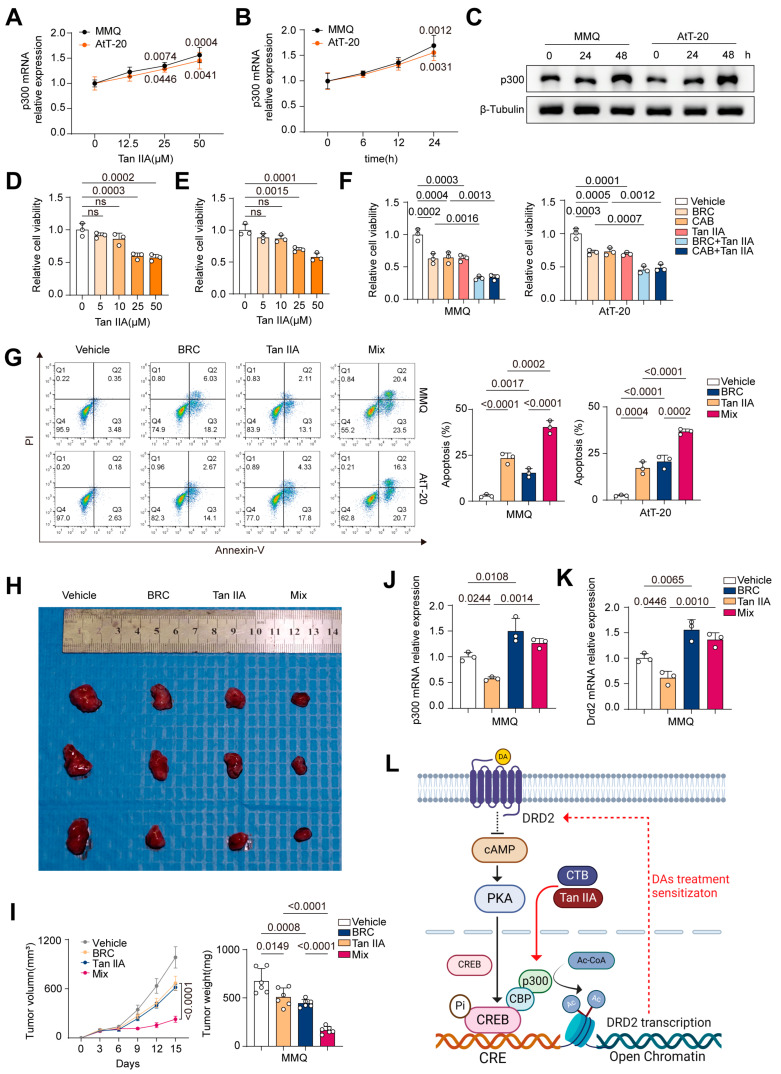
Tanshinone IIA upregulates p300 and synergizes with BRC to exert anti-tumor effects in pituitary tumors. (**A**–**C**) q-PCR of p300 expression in MMQ (**A**) and AtT-20 (**B**) cells treated with Tan IIA (0, 12.5, 25, 50 μM) for 24 h. (**C**) Western Blot (WB) analysis of p300 expression in MMQ and AtT-20 cells treated with Tan IIA (25 μM) for various periods (0, 24, and 48 h), *n* = 3. (**D**,**E**) Cell viability in MMQ (**D**) and AtT-20 (**E**) cells was measured using the CCK-8 assay after treatment with different concentrations of Tan IIA (0, 5, 10, 25, 50 μM) for 48 h, *n* = 3. (**F**) CCK-8 assay of cell viability in MMQ (**A**) and AtT-20 (**B**) cells treated with BRC (10 μM), CAB (25 μM), Tan IIA (25 μM), BRC (10 μM) + Tan IIA (25 μM), or CAB (25 μM) + Tan IIA (25 μM) for 48 h, *n* = 3. (**G**) Annexin-V apoptosis flow cytometry analysis of cell apoptosis in MMQ and AtT-20 cells treated with BRC (10 μM), Tan IIA (25 μM), or mix (BRC (10 μM) + Tan IIA (25 μM)) for 48 h, *n* = 3. (**H**,**I**) Subcutaneous tumor formation in nude mice using MMQ cells, followed by intraperitoneal injections of PBS, BRC (10 mg/kg/d), Tan IIA (20 mg/kg/d), or BRC (10 mg/kg/d) + Tan IIA (20 mg/kg/d) for 2 weeks, representative images of subcutaneous xenograft tumors (**H**), average volume of excised tumors ((**I**) left), average weight of excised tumors ((**I**) right). (**J**,**K**) MMQ cells were treated with BRC (10 μM), Tan IIA (25 μM), or mix (BRC (10 μM) + Tan IIA (25 μM)) for 48 h, q-PCR of p300 (**J**) and DRD2 (**K**) expression, *n* = 3. (**L**) A representative figure of the mechanism of action integrating the effects of DA, p300 and Tan IIA in the signaling pathway (figure created with Biorender.com). Data are shown as mean ± SD. Statistical analyses were conducted using one-way ANOVA. Abbreviations: DA, Dopamine agonist; BRC, Bromocriptine; CAB, Cabergoline; Tan IIA, Tanshinone IIA.

Tanshinone IIA (Tan IIA), a traditional Chinese medicine and a lipophilic component of Salvia miltiorrhiza, possesses neuroprotective, anti-inflammatory, antioxidant, and antitumor properties. Our experiments revealed that Tan IIA upregulates p300 expression in MMQ and AtT-20 cells (Figure 5A–C), suggesting that Tan IIA may exert antitumor effects on these cells by enhancing p300 expression in combination with BRC.

First, we assessed the effect of Tan IIA on the proliferation of both cell lines (Figure 5D,E). The results showed that 25 μM Tan IIA significantly inhibited proliferation in both cell lines after 48 h. Next, we treated the cells with 25 μM Tan IIA in combination with 10 μM BRC. The CCK-8 assay results (Figure 5F) indicated a synergistic inhibition of tumor proliferation, while flow cytometry analysis (Figure 5G) demonstrated a synergistic pro-apoptotic effect. In vivo subcutaneous xenograft experiments also confirmed (Figure 5H,I, Supplementary Figure S4A–E) that Tan IIA combined with BRC significantly inhibited tumor growth compared to BRC alone.

Finally, we examined the effect of Tan IIA and BRC, both individually and in combination, on the expression of p300 and DRD2 in both cell lines (Figure 5J,K, Supplementary Figure S4F–I). The results showed that the combination treatment upregulated p300 and DRD2 expression compared to the BRC group.

These findings provide a basis for the potential clinical application of Tan IIA combined with BRC in the treatment of pituitary adenomas.

## 3. Discussion

Prolactinomas often cause neurological dysfunction and reproductive disorders in patients due to their mass effect and excessive prolactin secretion. Treatment aims to reduce tumor size and inhibit prolactin hypersecretion [9]. Given the high expression of DRD2 on the tumor cell membrane, DA therapies such as BRC and CAB are considered first-line treatments [2]. However, 10–30% of patients exhibit resistance to DA therapy, showing high recurrence rates and negative impacts on quality of life [3]. Previous studies have identified several factors related to prolactinoma resistance, including reductions in epidermal growth factor (EGF) and nerve growth factor (NGF) [10,11], altered ratios of D2R long and short isoforms, high expression of androgen and estrogen receptors (AR and ER) [12,13,14], and dysfunctional microRNAs (miRNAs) [15]. The failure of DA therapy is primarily associated with decreased DRD2 expression in tumors. Research suggests that DRD2 expression may be regulated by epigenetic modifications, such as CpG island methylation in the DRD2 promoter region, changes in histone methylation and acetylation levels, which affect DRD2 transcription [6,16].

As a histone acetyltransferase, p300 is involved in activating various transcription factors and regulating tumor-related behaviors, including cell proliferation, migration, invasion, apoptosis, drug resistance, and metabolism. Highly homologous to CREB-binding protein (CBP), p300 often forms a transcriptional coactivator complex with CBP, which can be recruited to the nucleus by p-CREB. Numerous studies have demonstrated that p300-mediated acetylation of histone H3 at lysines 18 and 27 plays a crucial role in gene transcription regulation [17]. Based on these findings, we hypothesized that p300 may be associated with prolactinoma resistance and may play a role in regulating DRD2 transcription.

In clinical practice, we observed that some prolactinoma patients receiving bromocriptine (BRC) treatment fail to achieve normalized serum prolactin levels, with tumor size reductions remaining below 30%, indicating resistance to BRC therapy. To better understand the molecular characteristics of dopamine-resistant prolactinomas and to investigate the mechanisms underlying potential drug resistance during DA treatment, we established a clinical basis for subsequent in vivo and in vitro experiments. This approach allows for a more comprehensive exploration of the relationship between p300 and DA resistance, while also providing a foundation for strategies to enhance DA therapy efficacy. Given these considerations, our study initially analyzed clinical samples from 50 patients with bromocriptine-resistant prolactinomas, finding significantly lower p300 expression in relatively insensitive patient groups, suggesting a close association between p300 and DA resistance. Since the downregulation of the cAMP-PKA-CREB pathway constitutes a mechanism of DA treatment for pituitary adenomas [18], we confirmed that DA can downregulate p300 expression by inhibiting the cAMP-PKA-CREB pathway in cultured MMQ and AtT-20 cells, indicating that p300 downregulation is related to DA-mediated resistance.

p300 functions as an oncogenic factor in several cancers, such as gastric, esophageal squamous cell carcinoma (ESCC), lung, pancreatic, and prostate cancers [19,20,21,22,23]. However, in skin cancer, osteosarcoma, myelodysplastic syndrome (MDS)-related leukemia, and human papillomavirus (HPV)-positive head and neck squamous cell carcinoma (HNSCC), p300 plays a tumor-suppressive role [24,25,26,27]. To investigate the role of p300 in MMQ and AtT-20 cells, we activated the p300 HAT domain and observed increased cell proliferation. However, when combined with DA treatment, both cell lines exhibited significantly reduced proliferation, increased apoptosis, and inhibited subcutaneous tumor growth. These results indicate that p300 activation can enhance DA efficacy both in vitro and in vivo.

To further explore the mechanisms behind the synergistic effect of p300 activation combined with DA treatment for pituitary adenomas, we hypothesized that acetylation at H3K18/27, mediated by the p300 HAT domain, plays a critical role in regulating DRD2 transcription. We developed cell lines overexpressing either wild-type p300 or a histone acetyltransferase (HAT) mutant form of p300. Utilizing chromatin immunoprecipitation followed by quantitative PCR (ChIP-qPCR) and co-immunoprecipitation (Co-IP) assays, we found that acetylation of histone H3 at lysines 18 and 27 (H3K18/27ac) is enriched at the DRD2 promoter region and modulated by p300, with H3K27ac making a particularly significant contribution. These findings suggest that p300 regulates DRD2 transcription through H3K18/27 acetylation, providing deeper insight into the role of p300 as a histone acetyltransferase in DRD2 transcription regulation within resistant prolactinomas. This understanding offers a potential new strategy for addressing DA resistance.

Finally, we evaluated the potential of Tanshinone IIA (Tan IIA), a traditional Chinese medicine. As a primary lipophilic component of Salvia miltiorrhiza, Tan IIA has neuroprotective, anti-inflammatory, antioxidant, and antitumor effects. Previous studies have shown that Tan IIA can enhance tumor sensitivity to chemotherapy and radiotherapy [28]. Our experiments demonstrated that Tan IIA, in combination with bromocriptine, significantly inhibits MMQ and AtT-20 cell proliferation both in vitro and in vivo. This effect may be attributed to Tan IIA-mediated upregulation of p300 and DRD2 expression, supporting its potential as an effective strategy for combination therapy.

Additionally, the AtT-20 cell line, a model for pituitary corticotroph adenoma, was included in this study due to its expression of DRD2 receptors. Although AtT-20 cells differ from MMQ cells in subtype and hormone secretion profile, our experiments showed that DA activation of DRD2 inhibited the cAMP/PKA/CREB pathway in both cell lines, with a similar impact on p300 expression. Both cell lines exhibited comparable sensitization responses when combined with CTB or Tan IIA, suggesting that they provide a reasonable in vitro and in vivo model for studying dopamine-resistant prolactinomas. However, inherent differences between these cell types led to varied responses to DA treatment, with MMQ cells showing greater sensitivity to both DA monotherapy and combination treatments. This discrepancy may arise from differences in DRD2 receptor expression levels on the cell membrane and intracellular signaling regulation. Exploring these unique characteristics in the two tumor cell types could improve our understanding of resistance mechanisms in pituitary adenomas and offer valuable directions for future research.

## 4. Materials and Methods

### 4.1. Clinical Samples

Tumor specimens were collected from 50 prolactinoma patients who were resistant to bromocriptine treatment and underwent surgical intervention between January 2018 and January 2022 at the Department of Neurosurgery, Wuhan Tongji Hospital. Resistant specimens were defined as those from patients who, after receiving treatment with the maximum tolerated dose of dopamine agonist (DA) for at least 3–6 months, did not achieve serum prolactin levels within the normal range or at levels sufficient to restore normal gonadal function. Additionally, tumor reduction was less than 30% in overall size or less than 50% in the coronal plane [29]. Specimens meeting these criteria were collected as clinically resistant samples for this study. This study was approved by the Ethics Committee of Tongji Hospital (TJ-IRB20220325), and all patients provided informed consent. Based on the reduction in tumor maximum diameter and serum prolactin levels before and after bromocriptine treatment, the patients were divided into two groups: 25 relatively sensitive cases and 25 relatively insensitive cases.

### 4.2. Cell Culture, Treatment, and Transfection

Three cell lines were used in this experiment: pituitary tumor cell lines (MMQ, AtT-20) and 293T cells, all purchased from the American Type Culture Collection (ATCC, Manassas, VA, USA). MMQ cells were cultured in RPMI-1640 medium (G4535, ServiceBio, Wuhan, China), while AtT-20 and 293T cells were cultured in DMEM/High Glucose medium (G4612, ServiceBio, Wuhan, China). All media were supplemented with 10% fetal bovine serum (FBS, A5661701, Gibco, Thermo Fisher, Waltham, MA, USA) and 1% antibiotics (G4003, ServiceBio, Wuhan, China). Cells were maintained in a humidified incubator at 37 °C with 95% air and 5% CO_2_.

The drugs used in this study include bromocriptine (BRC, HY-12705A, MCE, Shanghai, China), cabergoline (CAB, HY-15296, MCE, Shanghai, China), forskolin (FSK, HY-15371, MCE, Shanghai, China), CTB (N-(4-chloro-3-trifluoromethyl-phenyl)-2-ethoxy-benzamide, HY-134964, MCE, Shanghai, China), tanshinone IIA (Tan IIA, HY-N0135, MCE, Shanghai, China), and phosphate-buffered saline (PBS, G4202, ServiceBio, Wuhan, China).

p300 overexpression and p300 HAT domain mutant plasmids were constructed by Tsingke Biological Technology, Beijing, China. The target sequences are shown in Supplementary Table S2.

Lentiviral transfection was carried out according to the protocols from our previous publication [30]. Briefly, 293T cells were seeded in 6-well plates, and 2 mL of DMEM medium containing 10% fetal bovine serum (FBS) was used to refresh the medium before transfection; the viral packaging plasmids (PSPAX, 1.25 μg and PMD2G, 1.25 μg) and target plasmids (NC-vector, 2.5 μg, p300-OE, 2.5 μg) were then added to 100 μL of serum-free DMEM/High Glucose medium. Simultaneously, 7.5 μL of transfection reagent (TL201, Lipomaster 2000 Transfection Reagent, Vazyme, Nanjing, China) was added to 100 μL of serum-free DMEM/High Glucose medium. After incubating the two mixtures for 5 min, they were added to the 293T cells. After 6–8 h, the medium was replaced with 2 mL of fresh DMEM containing 10% FBS. Viral supernatant was collected after 72 h, filtered using a 0.22 μm filter, and used to infect MMQ and AtT-20 cells. After 6–8 h, the viral supernatant was replaced with fresh medium, and the cells were selected with 5 mg/mL puromycin (S7417, Selleck, Shanghai, China) for 3 days to establish stable transfected cell lines.

### 4.3. Tissue Section Staining

Clinical and xenograft tumor specimens were prepared as 5 μm sections (BIOSSCI, Wuhan, China) for immunohistochemistry (IHC) and immunofluorescence (IF) staining analysis.

For IHC staining, clinical tumor tissue sections were rehydrated and blocked with 5% bovine serum albumin (BSA, GC305010, ServiceBio, Wuhan, China) at room temperature for 2 h. The sections were then incubated with anti-p300 antibody (sc-48343, Santa Cruz, TX, USA) at 4 °C for 16 h. Endogenous peroxidase activity was quenched with 3% H_2_O_2_, followed by incubation with biotinylated anti-mouse IgG (SV0002, Boster, Wuhan, China) at room temperature for 2 h. The sections were further incubated with a streptavidin-biotin complex (SV0002, SABC, Boster, Wuhan, China) for 30 min and visualized using DAB (BP0550, BP0770, DAB substrate:DAB chromogen = 1:20). Hematoxylin was used for nuclear counterstaining.

For IF staining, xenograft tumor sections were rehydrated and blocked with 5% BSA (GC305010, ServiceBio, Wuhan, China) at room temperature for 2 h. The sections were then incubated with anti-Ki-67 antibody (27309-1-AP, Proteintech, Wuhan, China) or anti-p300 antibody (sc-48343, Santa Cruz, TX, USA) at 4 °C for 16 h. This was followed by incubation with anti-rabbit IgG (GB25303, ServiceBio, Wuhan, China) or anti-mouse IgG (GB25301, ServiceBio, Wuhan, China) at room temperature for 2 h. Nuclei were stained with 4′,6-diamidino-2-phenylindole (DAPI, G1012, ServiceBio, Wuhan, China).

All stained sections were observed and imaged using a microscope (Olympus, Tokyo, Japan), and analyzed using Image J (version 1.53f51). All images were captured at 400× magnification under an optical microscope. Experiments were independently repeated six times.

### 4.4. Western Blotting

MMQ and AtT-20 cells were lysed using RIPA lysis buffer (G2002, Servicebio, Wuhan, China) or a nuclear and cytoplasmic protein extraction kit (P0028, Shanghai, China) containing phenylmethanesulfonyl fluoride (G2008, Servicebio, Wuhan, China) and phosphatase inhibitors (G2007, Servicebio, Wuhan, China). Protein concentrations were determined using a BCA assay kit (G2026, Servicebio, Wuhan, China). Loading buffer (G2075, Servicebio, Wuhan, China) was added to the lysates, followed by boiling for 15 min. The protein lysates were separated by SDS-PAGE on 6% or 10% gels and transferred onto PVDF membranes (IPFL00005, Millipore, Burlington, MA, USA). The membranes were blocked with Fast Blocking Buffer (G2052, ServiceBio, Wuhan, China) at room temperature for 15 min. The membranes were incubated with primary antibodies at 4 °C for 16 h, including E1A-associated protein p300 (p300, sc-48343, Santa Cruz, TX, USA), PKA-catalytic subunits (PKA-c, ab59218, Cambridge, UK), total cAMP response element-binding protein (t-CREB, ab32515, Abcam, Cambridge, UK), phosphorylated cAMP response element-binding protein (p-CREB, ab32096, Abcam, Cambridge, UK), CREB-binding protein (CBP, A26780, Abclonal, Wuhan, China), Histone H3 (H3, A17562, Abclonal, Wuhan, China), histone H3 lysine 18 acetylation (H3K18ac, A7257, Abclonal, Wuhan, China), histone H3 lysine 27 acetylation (H3K27ac, A22264, Abclonal, Wuhan, China), dopamine receptor D2 (DRD2, A12930, Abclonal, Wuhan, China), β-Tubulin (A12289, Abclonal, Wuhan, China), and β-Actin (AC038, Abclonal, Wuhan, China). After washing, the membranes were incubated with HRP-conjugated anti-rabbit or anti-mouse antibodies (AS014, AS003, Abclonal, Wuhan, China) at room temperature for 2 h. The target proteins were visualized and imaged using an ECL chemiluminescence kit (P10060, NCM Biotech, Suzhou, China) on a GeneGnome GRQ system (Syngene, Cambridge, UK). Experiments were independently repeated three times.

### 4.5. Co-Immunoprecipitation (co-IP)

Proteins were extracted from MMQ and AtT-20 cells using the IP lysis buffer (G2038, Servicebio, Wuhan, China) containing phosphatase inhibitors (G2007, Servicebio, Wuhan, China), and protein concentrations were determined using a BCA assay kit (G2026, Servicebio, Wuhan, China). Protein samples were incubated with anti-p-CREB or anti-p300 antibodies bound to Protein A/G Magnetic Beads (HY-K0202, MCE, Shanghai, China) at 4 °C for 16 h. The beads were magnetically separated to isolate proteins bound to p-CREB or p300, followed by elution and separation by SDS-PAGE. Western blotting was performed on the immunoprecipitated samples using antibodies against p-CREB, p300, CBP, H3K18ac, and H3K27ac. Experiments were independently repeated three times.

### 4.6. ChIP-qPCR

DNA from MMQ and AtT-20 cells was collected using the SimpleChIP^®^ Plus Enzymatic Chromatin IP Kit (Magnetic Beads) (#9005, CST, Danvers, MA, USA). Cells were first crosslinked with 1% formaldehyde to fix proteins to DNA, followed by cell lysis and chromatin digestion. The digested chromatin was diluted, and specific antibodies (H3, H3K18ac, H3K27ac) bound to Protein A/G Magnetic Beads (HY-K0202, MCE, Shanghai, China) were added to each IP sample. Samples were incubated at 4 °C for 16 h, then washed using a magnetic separation rack. Chromatin was eluted from the Antibody/Protein G Magnetic Beads and reverse cross-linked. DNA was then purified using spin columns. Real-time quantitative PCR was performed using SimpleChIP^®^ Universal qPCR Master Mix (#88989, CST, Danvers, MA, USA). The efficiency of the IP was calculated using the percentage of input method. The primer sequences are shown in Supplementary Table S3. Experiments were independently repeated three times.

### 4.7. Cell Apoptosis Assay

MMQ and AtT-20 cells were seeded in 6-well plates at a density of 2 × 10^5^ cells per well. After 48 h of treatment with various reagents, AtT-20 cells were digested with 0.25% trypsin without EDTA (G4011, Servicebio, Wuhan, China), and cell suspensions from both MMQ and AtT-20 cells were collected. The cells were centrifuged at 300× *g* for 5 min at 4 °C. The collected cells were stained with Annexin V-Alexa Fluor 647 and PI (40304ES50, Yeasen, Shanghai, China) for 15 min. Apoptosis levels were analyzed by flow cytometry, and data analysis was performed using FlowJo 10.8.1. Experiments were independently repeated three times.

### 4.8. Cell Viability Assay

Cell viability in different treatment groups was assessed using the CCK-8 assay (G4103, Servicebio, Wuhan, China). MMQ and AtT-20 cells were seeded in 96-well plates at a density of 5000 cells per well. After 48 h of treatment with various reagents, 100 µL of DMEM or RPMI-1640 medium mixed with 10 µL of CCK-8 reagent was added to each well. The cells were incubated for 1–2 h, and absorbance was measured at 450 nm using the Infinite^®^ F50 plate reader (Tecan, Männedorf, Switzerland) to calculate cell viability. Experiments were independently repeated three times.

### 4.9. Enzyme-Linked Immunosorbent Assay (ELISA)

The levels of prolactin (PRL) and adrenocorticotropic hormone (ACTH) in the supernatant were measured to reflect the secretory capacity of MMQ and AtT-20 cells, respectively. After drug treatment, the culture media from MMQ and AtT-20 cells were collected and centrifuged at 1000× *g* for 10 min at 4 °C to collect the supernatants. PRL levels were measured using the Rat PRL ELISA Kit (E-EL-R3006, Elabscience, Wuhan, China), and ACTH levels were measured using the Mouse ACTH ELISA Kit (E-EL-M0079, Elabscience, Wuhan, China).

After drug treatment, MMQ and AtT-20 cells were centrifuged at 1000× *g* for 10 min at 4 °C and resuspended in 1 mL PBS. The cells were then lysed by sonication. Intracellular cAMP levels were measured using the cAMP ELISA Kit (E-EL-0056, Elabscience, Wuhan, China). Intracellular PKA activity was detected using the PKA Kinase Activity Assay Kit (ab139435, Abcam, Cambridge, UK). Intracellular Ac-CoA ELISA Kit (E-EL-0125, Elabscience, Wuhan, China) Absorbance was measured at 450 nm using the Infinite^®^ F50 plate reader (Tecan, Männedorf, Switzerland), and PRL, ACTH, cAMP concentrations, and PKA kinase activity were calculated based on the standard curves. Experiments were independently repeated three times.

### 4.10. Real-Time Quantitative Polymerase Chain Reaction (qPCR)

Total RNA was extracted from MMQ, AtT-20 cells, or tumor tissues using the TransZol Up Plus RNA Kit (ER501-01-V2, Beijing, China). The RNA was reverse transcribed into cDNA using the HiScript^®^ II QRT SuperMix for qPCR (+gDNA wiper) (R223-01, Vazyme, Nanjing, China). qPCR was performed using the ChamQ Blue Universal SYBR qPCR Master Mix (Q312-03, Vazyme, Nanjing, China) on an ABI QuantStudio Real-Time PCR System (Thermo Fisher, Waltham, MA, USA). GAPDH was used as the internal control, and relative expression levels were calculated using the 2^∆∆CT^ method. The primers used in this study were purchased from Tsingke Biotechnology, and the primer sequences are shown in Supplementary Table S2. Experiments were independently repeated three times.

### 4.11. In Vivo Xenograft Assay

The animal research was approved by the Animal Ethics Committee of Tongji Hospital, Huazhong University of Science and Technology (TJH-202206015). The subcutaneous xenograft tumor model in nude mice was established according to the protocols from our previous publication [30]. Briefly, Male BALB/c nude mice, aged 6–8 weeks (Gempharmatech, Nanjing, China) and housed in the animal facility at Tongji Hospital, were randomly divided into four or six groups. MMQ or AtT-20 cells (1 × 10⁷ cells/100 µL) were subcutaneously injected into the right axilla of each mouse. Intraperitoneal injections were administered every two days with the following drugs: BRC (10 mg/kg/day), CAB (15 mg/kg/day), CTB (20 mg/kg/day), or Tan IIA (20 mg/kg/day). Tumor size was measured regularly, and tumor volume was calculated using the following formula: 0.5 × longest diameter × (shortest diameter)^2^. On day 15, all mice were sacrificed. Tumors were surgically excised, weighed, and fixed in 4% paraformaldehyde (G1101, Servicebio, Wuhan, China) overnight, followed by paraffin embedding and the preparation of 5 μm thick sections for further staining. Experiments were independently repeated six times.

### 4.12. Statistical Analysis

Statistical analyses were performed using GraphPad Prism (version 8.0.2). Data are presented as mean ± standard deviation (SD). The normality of data distribution was assessed using the Shapiro–Wilk test (n ≤ 50) for both clinical samples and cell line data. For normally distributed data, Pearson’s correlation analysis was used to assess the correlation between two variables. Unpaired *t*-tests were used for comparisons between two groups, while one-way analysis of variance (ANOVA) was applied for multiple group comparisons. *p*-values of less than 0.05 was considered statistically significant (represented as **** *p* < 0.0001; *** *p* < 0.001; ** *p* < 0.01; * *p* < 0.05).

## 5. Conclusions

Our study reveals the crucial role of p300 in DA-mediated resistance in prolactinomas and its regulation of DRD2 transcription through H3K18/27 acetylation. These findings provide new insights into the mechanisms of DA-resistant prolactinomas and establish a theoretical foundation for p300 activation as a therapeutic approach, supporting the clinical application of Tan IIA to enhance DA efficacy.

## Data Availability

The data presented in this study are available on request from the corresponding author.

## Supplementary Materials

The following supporting information can be downloaded at: https://www.mdpi.com/article/10.3390/ijms252312483/s1.
