# Supplementary material for: The Activation of p300 Enhances the Sensitivity of Pituitary Adenomas to Dopamine Agonist Treatment by Regulating the Transcription of DRD2
Source: Int J Mol Sci. 2024 Nov 21;25(23):12483. doi: 10.3390/ijms252312483 (PMC11641041; doi:10.3390/ijms252312483)
Supplement: Supplementary file 1 [file ijms-25-12483-s001.zip › ijms-3289906-supplementary figure legend.pdf]

Supplementary figure legend

Supplementary figure S1. DA downregulates p300 expression in pituitary tumor cells.

(A) 50 patients with drug-resistant prolactinomas were classified into a relatively sensitive group (n = 25) and a relatively insensitive group (n = 25), q-PCR of p300 expression in the two patient groups. (B-D) Subcutaneous tumor formation in nude mice using AtT-20 cells, followed by intraperitoneal injections of PBS, BRC (10 mg/kg/d), or CAB (15 mg/kg/d) for 2 weeks, q-PCR of p300 expression in AtT-20 (C) tumor tissues, immunofluorescence (IF) staining of Ki-67 expression in AtT-20 tumor tissue sections, representative images (B), scale bar, 50 μm, quantification (D), n = 6. Data were shown as mean ± SD. Statistical analyses were conducted using unpaired t-test, and one-way ANOVA. Abbreviations: BRC, Bromocriptine; CAB, Cabergoline.

Supplementary figure S2. Activation of p300 HAT activity synergizes with DA to exert anti-proliferative effects in pituitary tumors both in vitro and in vivo.

(A) AtT-20 cells were treated with BRC (10 μM), CAB (25 μM), CTB (50 μM), BRC (10 μM) + CTB (50 μM), or CAB (25 μM) + CTB (50 μM) for 48 hours, (A) Annexin-V apoptosis flow cytometry analysis of cell apoptosis, quantification (right), n = 3. (B) Subcutaneous tumor formation in nude mice using AtT-20 cells, followed by intraperitoneal injections of PBS, BRC (10 mg/kg/d), CAB (15 mg/kg/d), CTB (20 mg/kg/d), BRC (10 mg/kg/d) + CTB (20 mg/kg/d), or CAB (15 mg/kg/d) + CTB (20 mg/kg/d) for 2 weeks, representative images of subcutaneous xenograft tumors (left), average volumn of excised tumors (middle), average weight of excised tumors (right). (C) Immunofluorescence (IF) staining of Ki-67 expression in tumor tissue sections, scale bar, 50 μm, quantification (right), n = 6. Data were shown as mean ± SD. Statistical analyses were conducted using one-way ANOVA. Abbreviations: BRC, Bromocriptine; CAB, Cabergoline; CTB, N-(4-chloro-3-trifluoromethyl-phenyl)-2-ethoxy-benzamide); ns, not significant.

Supplementary figure S3. p300 promotes DRD2 transcription by increasing

histone H3K18/27 acetylation.

(A) MMQ and AtT-20 cells were treated with BRC (10 μM) for various periods (0, 12, 24, 48 hours), The acetyl-CoA ELISA kit was used to measure intracellular acetyl-CoA levels in MMQ and AtT-20 cells, n=3. (B) The general structure of wild-type p300 (WT-p300) and mutant p300 without the HAT domain (Mut-p300). (C) Subcutaneous tumor formation in nude mice using MMQ and AtT-20 cells, followed by intraperitoneal injections of PBS, BRC (10 mg/kg/d), CAB (15 mg/kg/d), CTB (20 mg/kg/d), BRC (10 mg/kg/d) + CTB (20 mg/kg/d), or CAB (15 mg/kg/d) + CTB (20 mg/kg/d) for 2 weeks, Western Blot (WB) analysis of DRD2 expression, n=6. Data were shown as mean ± SD. Statistical analyses were conducted using one-way ANOVA. Abbreviations: BRC, Bromocriptine; CAB, Cabergoline; CTB, N-(4-chloro-3-trifluoromethyl-phenyl)-2-ethoxy-benzamide); DRD2, Dopamine receptor D2.

Supplementary figure S4. Tanshinone IIA upregulates p300 and synergizes with BRC to exert anti-tumor effects in pituitary tumors.

(A) Subcutaneous tumor formation in nude mice using AtT-20 cells, followed by intraperitoneal injections of PBS, BRC (10 mg/kg/d), Tan IIA (20 mg/kg/d), or BRC (10 mg/kg/d) + Tan IIA (20 mg/kg/d) for 2 weeks, representative images of subcutaneous xenograft tumors (left), average volumn of excised tumors (middle), average weight of excised tumors (right). (B-E) Immunofluorescence (IF) staining of Ki-67 expression in MMQ and AtT-20 tumor tissue sections, representative images (B, C), scale bar, 50 μm, quantification (D, E), n = 6. (F-G) MMQ cells were treated with BRC (10 μM), Tan IIA (25 μM), or mix (BRC (10 μM) + Tan IIA (25 μM)) for 48 hours, q-PCR of p300 (F) and DRD2 (G) expression, n=3. (H-I) Western Blot (WB) analysis of DRD2 expression in MMQ and AtT-20 tumor tissues, n=6. Data were shown as mean ± SD. Statistical analyses were conducted using one-way ANOVA. Abbreviations: DA, Dopamine agonist; BRC, Bromocriptine; CAB, Cabergoline; Tan IIA, Tanshinone IIA;
