# Supplementary material for: The Activation of p300 Enhances the Sensitivity of Pituitary Adenomas to Dopamine Agonist Treatment by Regulating the Transcription of DRD2
Source: Int J Mol Sci. 2024 Nov 21;25(23):12483. doi: 10.3390/ijms252312483 (PMC11641041; doi:10.3390/ijms252312483)
Supplement: Supplementary file 1 [file ijms-25-12483-s001.zip › ijms-3289906-supplementary Table S1.pdf]

Baseline characteristics of 50 patients with prolactin-secreting pituitary adenomas.

Variables	Patients (n=50)
Gender, male/female (%)	22 (44) / 28 (56)
Age, years (mean ± SD)	31.0 ± 8.1
Pre-operation tumor diameter, cm (mean ± SD)	2.0 ± 0.9
Pre-operation PRL levels, ng/ml (mean ± SD)	951.2 ±456.1
Bromocriptine dose, mg/d (mean ± SD)	7.6 ± 4.2
Bromocriptine use time, months (mean ± SD)	7.2 ± 6.7
Follow-up time, months (mean ± SD)	59.8 ± 9.8
Clinical symptom (%)	
Headache	38 (76)
Diminution of vision	27 (54)
Menstrual disorder	42 (84)
Galactosis	18 (36)
Hyposexuality	12 (24)

Treatment efficacy analysis of 50 prolactin-secreting pituitary adenomas.

	relatively sensitive	relatively insensitive	*P* value
Total (n)	25	25	
Gender, male/female (%)	10(40)/15(60)	12(48)/13(52)	0.2545[a]
Age, years (mean ± SD)	29.4 ± 8.2	32.5 ± 7.6	0.1687[b]
Tumor maximum diameter before bromocriptine treatment, cm (mean ± SD)	2.3 ± 0.9	1.8 ± 0.9	0.0770[b]
Tumor maximum diameter after bromocriptine treatment, cm (mean ± SD)	1.8 ± 0.7	1.6 ± 0.8	0.4430[b]
Proportion of tumor diameter reduction after bromocriptine treatment, % (mean ± SD)	20.8 ± 4.0	9.3 ± 3.1	<0.0001[b]
PRL levels before bromocriptine treatment, ng/ml (mean ± SD)	834.1 ± 419.0	1068.0±469.6	0.0691[b]
PRL levels after bromocriptine treatment, ng/ml (mean ± SD)	412.8±225.4	763.0±342.3	<0.0001[b]
Proportion of PRL levels reduction after bromocriptine treatment, % (mean ± SD)	50.1±11.4	27.9±11.9	<0.0001[b]

[a] is Chi-square test

[b] is unpaired t test

PRL, prolactin.
