# Supplementary material for: The Activation of p300 Enhances the Sensitivity of Pituitary Adenomas to Dopamine Agonist Treatment by Regulating the Transcription of DRD2
Source: Int J Mol Sci. 2024 Nov 21;25(23):12483. doi: 10.3390/ijms252312483 (PMC11641041; doi:10.3390/ijms252312483)
Supplement: Supplementary file 1 [file ijms-25-12483-s001.zip › ijms-3289906-supplementary Table S2.pdf]

Rat Ep300 overexpression sequence:

TCTAGAATGGCCGAGAATGTGGTGGAACCCGGGCCGCCTTCAGCCAAGCG
GCCTAAACTCTCATCTCCGGCCCTCTCGGCGTCCGCCAGCGATGGCACAG
ATTTTGGTTCACTGTTTGACCTGGAACATGACTTACCAGATGAATTAATCA
ACTCAACAGAATTGGGACTAACCAATGGTGGTGATATTAGTCAGCTTCAG
ACAAGTCTTGGCATAGTACAAGATGCAGCCTCCAAACATAAACAACTGTC
AGAATTGCTAAGGTCTGGTAGTTCCCCCAACCTCAATATGGGAGTTGGTG
GCCCAGGCCAAGTTATGGCCAGCCAGGCCCAACAGAATAGCCCTGGATTA
AGTTTGATAAATAGCATGGTCAAAAGCCCAATGGCACAGACAGGCTTGAC
TTCTCCCAATATGGGGATGGGCAGTAGTGGACCAAATCAGGGTCCTACTC
AGTCCACAGCAGGTATGATGAACAGCCCAGTGAATCAACCTGCCATGGGA
ATGAACACAGGGATGAATGCTGGCATGAATCCTGGAATGTTGGCTGCAGG
CAATGGACAAGGGATAATGCCCAATCAAGTCATGAATGGTTCAATTGGAG
CAGGCCGGGGAAGGCCAAACATGCAGTACCCAAATGCAGGCATGGGCAA
TGCTGGCAGTTTATTGACTGAGCCACTACAGCAGGGCTCTCCTCAGATGG
GAGGACAACCAGGATTGAGAGGCCCCCAGTCACATAAGATGGGAATGAT
GAGCAATCCCACTCCTTATGGTTCACCATACACTCAGAATTCTGGACAGC
AGATTGGAGCAAGTGGCCTTGGTCTCCAAATTCAGACAAAGACTGTTTTA
CCAAATAACCTTTCTCCATTTGCAATGGACAAAAAAGCAGTTACTGGTGG
GGGAATGCCCAACATGGGCCAACAGCCTACCCCGTCGGTCCAACAGCCAG
GCCTGGTGAATCCGGTTGCCCCAGGGATGGGTTCTGGAGCGCATACAGCT
GATCCAGAGAAGCGCAAACTCATCCAGCAGCAGCTTGTTCTCCTTTTACAT
GCTCACAAGTGCCAGCGCCGGGAGCAAGCTAATGGAGAAGTGAGGCAGT
GCAATCTTCCCCACTGTCGCACCATGAAAAATGTTCTAAACCATATGACA
CATTGTCAGTCAGGCAAATCCTGTCAAGTGGCACATTGTGCGTCTTCTCGA
CAAATCATTTCACACTGGAAAAATTGCACAAGGCATGATTGTCCTGTGTG
TCTTCCTCTCAAAAATGCTGGGGATAAGCGAAATCAACAGTCAATTTTGA
CTGGAGCACCAGTCGGGCTTGGAAACCCTAGCTCTCTAGGAGTGGGACAG
CAGTCCACCCCTAGCCTAAGCACTGTCAGTCAGATTGATCCCAGCTCTATA
GAGCGAGCTTATGCTGCTCTCGGACTACCCTATCAAGTAAACCAGATTCC
ACCACAACCCCCCGTCCAAGCTAAGAATCAACAAAGTCAGCCGTCTGGAC
AGTCTCCCCAAGGCATGCGGTCCATGAACAACATGAGTGCCAGTCCTATG
GGTGTAAATGGAGGTGTAGGGGTTCAGACACCGAATCTTCTTTCTGACTC
CATGTTGCATTCAACCATAAATTCTCAGAACCCAATGATGAATGAAAATG
CCAGTGTGGCCTCTCTGGGTCCTTTGCCCACAGCAGCCCAGCCATCTAGTA
CTGGAATTCGAAAACAGTGGCATGAAGATATTACTCAGGATCTTCGAAAC
CACCTTGTTCATAAACTTGTTCAAGCCATATTTCCCACTCCCGATCCTGCT
GCTTTAAAAGACCGACGGATGGAAAACCTTGTTGCATATGCTCGTAAAGT
AGAAGGGGACATGTATGAATCTGCAAACAATAGAGCGGAATATTACCAC
CTCTTAGCTGAGAAGATCTATAAGATCCAGAAAGAACTAGAAGAGAAAC
GAAGGACTAGACTACAGAAACAAAACATGCTACCAAGTGCTCCTGGCATG
GTTCCAGTGCCTATGAATACTGCACCTAACATGGGACAGCAACCAACAGG

AATGACTACCAATGGTCCTCTACCTGATCCATCTATGATCCGTGGCAGTGT
GCCAAACCAGATGATGCCTCGGATGACACCACAACCTGGTTTGAACCAGT
TTGGACAGATGAATATACCACAGCCCCCTATTGGGCCCCGGCAGCCGTCT
CCTCTCCAGCATCACGGACAGCTGGCCCAGTCGGGCTCACTCAATCCGCC
TATGGGCTATGGACCTCGCATGCAGCAGACTTCTGGCCAGAACCAATTTC
TCTCCCAGACTCAGTTTCCATCCCAAGGAATGAATGTAACAAACATGCCTT
TGGCTCCATCCAGCAGTCAAGCACCAGTGTCTCAAGCACAGATGTCCAGT
TCTTCCTGCCCAGTGAACTCTCCTATAATGCCTCCAGGGTCTCAAGGGAGC
CATATTCACTGTCCTACTCTGCCTCAACAAGCTCATCAGAATTCACCTTCT
CCTGTTCCTAGCCGTACCCCTACTCCTCATCACACACCCCCAAGCATAGGG
ACTCAACAACCAGCAGCAACAGCAATTCCAGCGCCTGTTCCTACACCCCC
TGCCATGCCGCCTGGAGCACAGCCCCAGTCTCTACGCCCCCCATCTAGAC
AGACTCCTACACCACCAACACATCTGCCTCCCCAAGTGCAACCTTCACTTC
CTGTTCCTCCTGCTGACCAGTCCCAGCCACAGCCTCGCTCACAGCAGAGC
ACAGCAGCTTCTGTCCCCACCCCAACAGCACCACTGCTGCCTCCTCAGCCT
CCTACTCCACTTTCTCAGCCAGCTGTAAGTGTTGAAGGCCAGGTGTCAAAC
CCTCCATCTACTAGTAGCACAGAAGTGAATTCTCAGACTATTCCTGAGAA
GCAACCATCACAGGAAGTCAAAATGGAGGTTAAAATGGACGTGGATCAA
CCAGAACCTGCAGATGCTCAACCTGATGACACAAAGGAGGCTAAAGCTG
AGGATGGTAAAGTAGAACCTACAGAAACAGAGGAGAGAGGCCCTGAGTT
AAAGACTGAAGTGAAAGAGGAGGAGGACCAGCCGAGCACCTCTGCTGCA
CAGTCCTCCCCTGCTCCTGGACAGTCAAAGAAGAAGATTTTCAAACCAGA
AGAATTACGGCAGGCTCTGATGCCAACATTGGAGGCACTTTACCGACAGG
ACCCAGAATCCCTTCCCTTTCGTCAGCCTGTGGACCCGCAGCTTCTAGGAA
TCCCTGATTACTTTGATATTGTGAAGAGCCCCATGGATCTTTCTACAATCA
AGAGGAAGTTAGATACTGGACAGTATCAAGAGCCCTGGCAATATATAGAT
GATATTTGGCTTATGTTCAACAATGCTTGGTTATATAACCGTAAAACTTCA
AGGGTATACAAATATTGCTCCAAACTTTCTGAAGTATTTGAACAAGAAAT
TGACCCTGTCATGCAAAGCCTTGGCTACTGCTGTGGCAGAAAGTTGGAGT
TCTCTCCACAGACTCTGTGTTGCTATGGCAAACAGTTATGCACAATCCCCC
GTGATGCTACTTACTACAGTTACCAGAACAGGTATCATTTCTGTGAGAAGT
GTTTCAATGAAATCCAAGGGGAGAGCGTTTCTTTGGGTGATGACCCTTCCC
AACCTCAAACTACAATAAATAAAGAACAATTTTCCAAGAGAAAAAATGA
CACACTGGATCCAGAACTGTTTGTTGAGTGCACAGAGTGTGGAAGAAAGA
TGCACCAAATCTGTGTCCTTCACCATGAGGTCATCTGGCCATCTGGGTTTG
TCTGTGATGGCTGTTTAAAGAAAACTGCACGAACTAGGAAAGAAAATAAG
TTTTCTGCTAAAAGATTGCCTTCTACCAGACTTGGGACCTTTCTGGAGAAT
CGAGTGAATGACTTTCTGAGGCGACAAAATCACCCTGAATCAGGAGAGGT
CACTGTTCGGGTTGTTCATGCTTCTGACAAAACTGTGGAGGTGAAACCAG
GCATGAAAGCAAGGTTTGTAGATAGTGGAGAGATGGCAGAATCTTTTCCA
TATCGAACCAAGGCCCTGTTTGCCTTTGAAGAAATTGATGGTGTTGACTTG
TGTTTCTTCGGCATGCATGTTCAAGAATATGGCTCTGACTGTCCCCCTCCC
AACCAGAGGAGAGTATACATATCTTACCTCGATAGTGTTCATTTCTTCCGT
CCTAAATGCTTGCGGACTGCAGTCTATCATGAAATTCTAATTGGATATTTG

GAGTATGTCAAGAAATTAGGATACACAACAGGGCATATCTGGGCCTGTCC
ACCAAGTGAAGGAGATGACTATATCTTCCATTGCCATCCACCCGATCAGA
AGATACCAAAGCCCAAGCGCCTGCAAGAATGGTACAAAAAGATGCTTGA
CAAGGCTGTGTCAGAACGCATTGTCCATGACTACAAGGATATTTTAAAGC
AAGCTACTGAAGATCGATTAACAAGTGCAAAGGAATTACCTTACTTTGAA
GGTGACTTCTGGCCCAATGTTCTGGAAGAAAGCATCAAGGAACTTGAACA
GGAAGAGGAAGAGAGGAAACGGGAAGAAAACACCAGCAACGAGAGTAC
CGATGTAACAAAAGGCGATAGCAAAAATGCTAAGAAGAAGAATAACAAG
AAGACCAGCAAAAACAAGAGCAGCCTGAGCAGGGGCAACAAGAAGAAG
CCTGGCGTGCCCAACGTGTCTAATGACCTTTCTCAGAAACTGTACGCCACC
ATGGAAAAGCATAAAGAGGTCTTCTTTGTAATCCGCCTCATCGCTTGTCCT
GCTCCCAACTCCCTGCCTCCCATTGTTGATCCTGACCCTCTCATCCCCTGC
GACCTGATGGATGGTAGAGATGCATTCCTCACCCTTGCAAGGGATAAGCA
CCTGGAATTCTCTTCACTTCGAAGAGCCCAGTGGTCTACCATGTGCATGCT
GGTGGAGCTGCACACACAGAGTCAAGATCGCTTTGTCTACACCTGCAATG
AGTGCAAGCACCACGTGGAGACACGCTGGCACTGCACTGTGTGTGAGGAT
TATGACCTGTGTATCACCTGTTACAACACTAAAAATCATGACCACAAAAT
GGAGAAATTAGGCCTGGGCTTGGATGACGAGAGCAACAACCAGCAAGCC
GCAGCCACACAGAGCCCAGGAGACTCTCGTCGCCTGAGCATCCAACGCTG
CATCCAGTCGCTGGTGCACGCCTGCCAGTGCCGCAATGCCAACTGCTCCCT
CCCTTCCTGCCAGAAGATGAAGAGGGTGGTGCAGCACACCAAAGGCTGCA
AACGGAAAACCAACGGTGGGTGCCCCATTTGCAAGCAGCTCATTGCCCTT
TGCTGCTACCATGCCAAGCACTGCCAGGAGAACAAGTGCCCGGTGCCGTT
CTGCCTCAACATCAAACAGAAGCTCCGGCAGCAGCAGCTGCAGCACCGGC
TCCAACAGGCTCAGATGCTGCGCAGGAGGATGGCCAGCATGCAACGGACT
GGGGTGGCGGGGCAGCAGCAGGGCCTGCCGTCCCCAACTCCTGCTACTCC
AACTACCCCAACTGGCCAACAGCCAGCCACCCCACAGACACCCCAGCCCC
AACCCACCTCTCAACCGCAGCCCACTCCTCCCAACAACATGACACCCTAC
TTACCCAGGACTCAAACTGCTGGCCCTGTGTCCCAGGGTAAGGCAGCAGG
CCAAGTAACCCCACCAACCCCACCTCAGACTGCTCAGCCCCCACTTCCAG
GGCCTCCACCCGCAGCAGTAGAGATGGCAATGCAGATTCAGAGGGCAGC
AGAGACACAGCGCCAGATGGCCCATGTACAAATTTTCCAAAGGCCAATCC
AGCACCAGATGGCACCTATGGCCCCTATGGGTATGAACCCACCTCCTATG
GCCAGAGGTCCTGGTGGACATTTGGATCCAGGAATGGGTCCCGCAGGAAT
GCAGCAGCAGCCACCTTGGGCCCAAGGAGGAATGCCTCAGCCCCAGCAG
ATGCAGTCAGGGATGCCAAGGCCAGCCATGATGTCAGTGGCCCAGCATGG
TCAGCCTTTGAACATGGCTCCACAGCCAGGATTGGGCCAAGTAGGTGTGA
GCCCTCTCAAGCCAGGCACTGTGTCTCAACAAGCCTTACAAAACCTTTTGC
GGACTCTCAGGTCTCCCAGTTCTCCCTTACAGCAGCAACAGGTGCTTAGTA
TCCTTCATGCCAACCCCCAACTGTTGGCTGCATTCATCAAGCAGCGGGCTG
CCAAGTATGCCAACTCTAATCCACAGCCTCTCCCTGGACAGCCTGGCATG
CCCCAGGGACAGCCAGGGCTGCAGCCACCGACCATGCCAGGTCAGCAAG
GTGTCCACTCCAACCCAGCCTTGCAGAACATGAATCCTATGCAGGCAGGA
GTCCAGAGAGCTGGCTTGCCTCAGCAGCAGCCTCAGCAGCAGCTCCAGCC

ACCCATGGGAGGGATGAGTCCCCAAGCTCAGCAAATGAACATGAATCAC
AACACCATGCCTTCACAGTTCAGAGACATCTTAAGACGTCAGATGATGCA
ACAGCAGGGAGCAGGGCCAGGAATTGGCCCTGGAATGGCCAACCACAAC
CAGTTCCAGCAGCCCCAAGGAATTGGCTATCCACCCCAACAGCAGCAGCA
GCAGCAACAGCAGCGAATGCAGCATCACATGCAGCAAATGCAGCAAGGA
AATATGGGACAGATGGGTCAGCTTCCCCAGGCTTTGGGGGCTGAGGCGGG
AGCCAGTCTACAGGCCTATCAGCAGCGACTCCTTCAGCAGCAGATGGGGT
CTCCTGCTCAGCCCAACCCTATGAGCCCACAGCAGCATATGCTCCCAAAT
CAGGCACAGTCCCCACACCTACAAGGTCAACAAATCCCTAATTCTCTCTCC
AATCAAGTGCGCTCTCCCCAGCCTGTCCCTTCTCCTCGACCACAGTCTCAG
CCCCCCCATTCCAGCCCGTCTCCGAGGATGCAGCCTCAGCCTTCTCCACAC
CATGTTTCTCCACAGACCAGTTCCCCACACCCTGGACTGGTAGCTGCCCAG
GCTGCCAACCCCATGGAACAAGGGCATTTTGCCAGCCCAGACCAGAATTC
AATGCTTTCACAGCTCGCTAGCAATCCAGGCATGGCAAACCTCCACGGTG
CAAGTGCCACGGACCTGGGACTGAGCGCCGATAGTGCAGACTTGAGTTCA
AACCTCTCACAGAGTACACTAGACATACACTAGCCCATATGGTGAGCGTG
GACTTTCCTGAAATGATGGCAGAGATCATCTCTGTGCAAGTGCCCAAGAT
CCTTTCTGGGAAAGTCAAGCCCATCTATTTCCACACACAGgactacaaagaccatga
cggtgattataaagatcatgacatcgattacaaggatgacgatgacaagTGAGCTAGC

Mouse Ep300 overexpression sequence:

TCTAGAATGGCCGAGAATGTGGTGGAACCCGGGCCGCCTTCAGCCAAGCG
GCCTAAACTCTCATCTCCGGCCCTCTCGGCGTCCGCCAGCGATGGCACAG
ATTTTGGTTCACTGTTTGACCTGGAACATGACTTACCAGATGAATTAATCA
ACTCTACAGAATTGGGACTAACCAATGGTGGCGATATCAGTCAGCTTCAG
ACAAGTCTTGGCATAGTACAAGATGCAGCCTCGAAACATAAACAGCTGTC
AGAACTGCTGAGGTCTGGTAGCTCCCCAAACCTCAACATGGGAGTCGGTG
GCCCAGGCCAAGCGATGGCCAGCCAGGCCCAACAGAACAGCCCTGGATT
AAGTTTGATAAATAGCATGGTCAAAAGCCCAATGGCACAGACAGGCTTGA
CTTCTCCAAACATGGGGATTGGCAGTAGTGGACCAAATCAGGGTCCTACT
CAGTCCCCAGCAGGTATGATGAACAGTCCAGTGAACCAGCCTGCCATGGG
AATGAACACAGGGATGAATGCTGGCATGAATCCTGGAATGTTGGCTGCAG
GCAATGGACAAGGGATAATGCCCAATCAAGTCATGAACGGTTCCATTGGA
GCAGGCCGGGGACGGCCAAACATGCAGTACCCAAATGCAGGCATGGGCA
ATGCTGGCAGTTTATTGACTGAGCCACTACAGCAGGGCTCTCCTCAGATG
GGAGGACAGCCAGGATTGAGAGGCCCCCAACCACTTAAGATGGGAATGA
TGAACAATCCCAGTCCTTATGGTTCACCATACACTCAGAATTCTGGACAGC
AGATTGGAGCAAGTGGCCTTGGTCTCCAAATTCAGACAAAGACTGTTCTA
CCAAATAACTTATCTCCATTTGCAATGGACAAAAAGGCAGTTCCTGGTGG
GGGAATGCCCAGTATGGGCCAGCAGCCTACCCCATCGGTCCAGCAGCCAG
GCCTGGTGACTCCAGTTGCCGCAGGAATGGGTTCTGGAGCACACACAGCT
GATCCAGAGAAGCGCAAGCTCATCCAGCAGCAGCTTGTTCTCCTTTTACAT
GCTCACAAGTGCCAGCGCCGGGAGCAAGCTAATGGGGAAGTGAGGCAGT

GCAACCTTCCTCACTGTCGTACCATGAAAAATGTCCTAAACCATATGACA
CATTGCCAGTCAGGCAAATCCTGCCAAGTGGCACATTGTGCATCTTCTCGA
CAAATCATTTCACACTGGAAAAATTGCACAAGGCATGATTGTCCTGTGTG
TCTTCCTCTCAAAAATGCTGGGGATAAGCGAAATCAACAGTCAATTTTGA
CTGGAGCACCAGTTGGGCTTGGAAACCCTAGCTCTCTAGGAGTGGGGCAG
CAGTCCACTCCTAGCCTAAGCACTGTTAGTCAGATTGACCCCAGCTCTATA
GAGCGAGCTTACGCTGCTCTTGGACTACCCTATCAAGTAAACCAGATTCC
ACCACAACCCCAGGTACAAGCAAAGAATCAACAAAGCCAGCCATCTGGA
CAGTCTCCCCAGGGCATGCGGTCTGTGAACAACATGAGTGCTAGTCCTAT
GGGTGTAAATGGAGGTGTTGGGGTTCAGACGCCAAATCTTCTTTCTGACTC
CATGTTGCATTCAACTATAAATTCTCAAAACCCAATGATGAGTGAAAATG
CTGGTGTGGCCTCCCTGGGTCCTTTGCCAACAGCAGCTCAGCCATCTAGTA
CTGGAATTCGAAAACAGTGGCATGAAGATATTACTCAGGATCTTCGAAAC
CACCTTGTTCATAAACTTGTTCAAGCCATATTTCCCACTCCGGATCCTGCT
GCTTTAAAAGACCGACGGATGGAAAATCTTGTTGCATATGCTCGTAAAGT
GGAAGGGGACATGTATGAATCTGCAAACAATAGAGCGGAATACTATCAC
CTCCTAGCCGAGAAGATCTATAAGATCCAGAAGGAACTAGAAGAGAAAC
GAAGGACTAGACTACAGAAACAGAACATGCTACCGAATGCTCCTGGCATG
GGTCCAGTTCCTATGAATACTGGGTCGAACATGGGGCAGCAACCAACAGG
AATGACTACCAATGGTCCTGTACCTGACCCGTCTATGATCCGTGGCAGTGT
GCCGAACCACATGATGCCTCGGATGACTCCACAGCCTGGTTTGAATCAAT
TTGGGCAGATGAATATGCCACAGCCCCCTATTGGACCCCGGCAACCCTCT
CCTCTTCAGCACCATGGACAATTGGCTCAGTCTGGGTCACTCAATCCGCCT
ATGGGCTATGGACCTCGCATGCAGCAGGCTTCCGGCCAGAACCAGTTCCT
CTCCCAGACTCAGTTCACATCCCAAGGAATGAATGTAACAAACATGCCTT
TGGCTCCATCCAGCGGTCAAGCACCAGTGTCTCAAGCACAAATGTCCAGT
TCTTCTTGCCCAGTGAACTCTCCTATAATGCCTCCAGGGTCTCAAGGGAGC
CACATTCACTGTCCTACTCTGCCTCAACAAGCTCATCAGAATTCACCTTCT
CCTGTTCCTAGCCGCACCCCTACTCCTCATCATACCCCCCCAAGCATAGGG
AATCAACCACCACCAGCAACAGCAATTCCAACACCTGTTCCTACACCTCC
TGCCATACCACCTGGACCACAGCCCCCATCTCTGCATCCCTCATCTAGACA
AACACCTACACCACCAACACATCTGCCTCCCCAAGTGCAGCCTTCACTTCC
TGCTGCTCCTTCTGCTGACCAGTCCCAGCAACAGCCTCGGTCACAGCAGA
GCACAGCAGTTTCTGTTCCTACCCCAACAGCACCATTGCTGCCTCCTCAGC
CTTCCACTCCGCTTTCTCAGCCAGCTGTGAGCATTGAAGGCCAGGTCTCAA
ACCCTCCATCTACTAGTAGCACCGAAGTGAATTCTCAGACCATTCCTGAG
AAGCAGCCTTCACAGGAAGTGAAAATGGAGTCTAAAATGGAGGTGGATA
AGCCAGAACCAGCAGATGCTCAACCTGAGGATACAAAGGAGGCTAAAGG
TGAGGATGTTAAAGTAGAACCTACAGAAATGGAGGAGAGAGGCCCTGAG
TTAAAAACTGATGGGAAAGAGGAGGAAGAACAGCCAAGCACCTCTGCTA
CCCAGTCCTCCCCAGCTCCTGGACAGTCAAAGAAGAAGATTTTCAAACCA
GAGGAATTACGACAGGCTCTGATGCCAACATTGGAGGCACTTTACCGGCA
GGACCCAGAATCTCTTCCCTTTCGTCAGCCTGTGGACCCGCAGCTTCTAGG
AATCCCTGATTACTTTGATATTGTGAAGAGCCCCATGGATCTTTCTACAAT

CAAGAGGAAGTTAGATACTGGACAATACCAAGAGCCCTGGCAATATATA
GATGATATTTGGCTTATGTTCAACAATGCCTGGTTATATAACCGTAAAACT
TCAAGGGTATACAAATATTGCTCTAAACTTTCTGAAGTATTTGAACAAGA
AATTGACCCTGTCATGCAAAGCCTTGGATACTGTTGTGGCAGAAAGTTGG
AGTTTTCTCCACAGACTCTTTGTTGCTATGGGAAACAGTTATGCACAATCC
CCCGTGATGCTACTTACTACAGTTACCAGAACAGGTATCATTTCTGTGAGA
AGTGTTTCAATGAAATCCAAGGGGAGAGCGTTTCTTTGGGTGATGACCCTT
CCCAACCTCAAACTACAATAAATAAAGAACAGTTTTCCAAGAGAAAAAAT
GACACGTTGGATCCTGAACTGTTTGTTGAGTGCACAGAGTGTGGAAGAAA
GATGCACCAGATCTGTGTCCTTCACCACGAGATCATCTGGCCATCTGGGTT
TGTCTGTGATGGCTGTTTAAAGAAAACTGCACGAACTAGGAAAGAAAATA
AGCTTTCTGCTAAAAGATTGCCATCTACCAGACTTGGGACCTTTCTGGAGA
ATCGAGTGAATGACTTTCTGAGGCGACAAAATCACCCTGAATCAGGAGAG
GTCACTGTTCGGGTTGTTCATGCTTCTGACAAAACTGTGGAAGTGAAACC
AGGCATGAAAGCAAGGTTTGTAGATAGTGGAGAGATGGCAGAATCTTTTC
CATACCGAACAAAGGCCCTGTTTGCCTTTGAAGAAATTGATGGTGTTGAC
TTGTGTTTCTTCGGCATGCATGTTCAAGAATATGGCTCTGACTGCCCCCCT
CCCAACCAGAGGAGGGTATACATATCTTACCTCGATAGTGTTCATTTCTTC
CGTCCTAAATGCTTGCGGACTGCAGTCTATCATGAAATTCTAATTGGATAT
TTGGAATATGTCAAGAAATTAGGATACACAACAGGGCACATCTGGGCCTG
TCCACCCAGTGAAGGGGATGACTATATCTTCCATTGCCATCCACCTGATCA
GAAGATACCAAAGCCCAAGCGCCTGCAAGAATGGTACAAAAAGATGCTT
GACAAGGCTGTATCAGAACGTATTGTCCATGACTACAAGGATATTTTAAA
ACAAGCTACTGAAGATCGATTAACAAGTGCAAAGGAACTACCCTACTTTG
AAGGTGATTTCTGGCCCAATGTTCTAGAAGAAAGCATCAAGGAGCTTGAA
CAGGAAGAGGAAGAGAGGAAACGGGAAGAGAACACCAGCAACGAGAGT
ACTGATGTAACAAAAGGAGACAGCAAAAATGCTAAGAAGAAGAATAACA
AGAAAACCAGCAAAAACAAGAGCAGCCTGAGTAGGGGCAATAAGAAGA
AGCCTGGTATGCCCAATGTGTCTAATGACCTTTCTCAGAAACTGTATGCCA
CCATGGAAAAGCATAAAGAGGTTTTCTTTGTGATCCGCCTCATCGCTTGTC
CTGCTCCCAATTCCCTGCCTCCCATTGTTGATCCTGACCCTCTCATCCCCTG
TGACCTGATGGACGGTAGAGATGCATTTCTCACCCTTGCAAGGGACAAGC
ACCTGGAGTTCTCTTCACTTCGAAGAGCCCAGTGGTCTACCATGTGCATGC
TGGTGGAGCTCCATACACAGAGTCAGGATCGCTTTGTCTACACCTGCAAC
GAGTGCAAGCACCACGTGGAGACACGCTGGCATTGCACTGTGTGTGAGGA
TTATGACCTGTGCATCACTTGTTATAACACTAAAAATCATGACCACAAAAT
GGAGAAACTAGGCCTTGGCTTAGATGATGAGAGCAATAACCAGCAAGCT
GCAGCTACACAGAGCCCAGGAGACTCCCGCCGCCTGAGCATCCAACGCTG
CATCCAGTCTCTGGTGCATGCCTGTCAGTGCCGCAACGCCAACTGCTCCCT
GCCTTCCTGCCAGAAGATGAAGAGGGTTGTGCAGCACACCAAAGGCTGCA
AACGGAAAACCAATGGTGGGTGCCCCATTTGCAAACAGCTTATTGCCCTC
TGCTGCTATCATGCCAAGCACTGCCAGGAGAACAAGTGCCCGGTGCCATT
TTGCCTCAACATCAAGCAAAAGCTCCGGCAGCAACAGCTGCAGCACCGGC
TCCAACAGGCTCAGATGCTCCGTAGGAGGATGGCCAGCATGCAACGAACT

GGTGTGGCGGGGCAGCAGCAGGGCCTGCCGTCCCCAACTCCTGCTACACC
AACTACCCCTACTGGCCAACAGCCAGCCACCCCACAGACACCCCAGCCCC
AACCCACCTCTCAACCACAGCCCACTCCTCCCAACAACATGACACCCTAC
TTACCCAGGACTCAGACTACTGGCCCTGTGTCCCAGGGTAAGGCACCAGG
CCAGGTGACCCCACCTACCCCACCTCAGACTGCTCAGGCCCCCCTTCCAG
GGCCTCCACCTGCAGCAGTAGAAATGGCAATGCAAATTCAGAGGGCAGC
AGAGACACAGCGCCAGATGGCCCATGTACAGATTTTCCAAAGGCCAATCC
AGCACCAGATGCCCCAGATGTCACCCATGGCCCCTATGGGCATGAACCCA
CCTCCTATGGCCAGAGGTCCTGGTGGGCATTTGGATCCAGGAATAGGTCC
AGCAGGAATGCAGCAACAGCCACCTTGGGCCCAAGGAGGAATGCCTCAG
CCCCAGCAGATGCAGTCAGGGATGCCAAGGCCAGCCATGATGTCAGTGGC
CCAGCATGGTCAGCCTTTAAACATGGCTCCACAACCAGGATTGGGCCAAG
TGGGTGTGAGCCCTCTCAAGCCAGGCACTGTGTCTCAACAAGCCTTACAA
AACCTTTTGCGGACTCTCAGGTCTCCCAGTTCTCCCTTACAGCAGCAACAG
GTGCTTAGTATCCTTCATGCCAACCCCCAACTGTTGGCTGCATTCATCAAG
CAGCGGGCTGCCAAGTATGCCAACCCTAATCCACAGCCTCTCCCTGGACA
GCCTGGCATGACCCAGGGACAGCCAGGGCTGCAGCCACCTACCATGCCTG
GCCAGCAAGGTGTCCACTCCAACCCAGCCTTGCAGAACATGAATCCTCTG
CAGGCAGGTGTCCAGAGAGCTGGCCTGCCCCAGCAGCAGCCGCAGCAGC
AGCTCCAGCCACCCATGGGAGCAATGAGTCCCCAAGCTCAGCAAATGAAC
ATGAATCACAATACCATGCCTTCACAGTTCAGAGACATCTTAAGACGGCA
GATGATGCAACAGCAGGGAGCAGGGCCAGGAATCGGCCCTGGAATGGCC
AACCAGTTCCAGCAGCCCCAAGGAATTGGCTATCCACCGCAGCAGCAGCA
GCAGCAGCGAATGCAGCATCACATGCAGCAAATGCAGCAAGGAAATATG
GGACAAATGGGTCAGCTTCCCCAGGCTCTGGGGGCTGAGGCAGGAGCCA
GTCTACAGGCCTATCAGCAGCGACTTCTTCAACAACAGATGGGGTCTCCT
GCTCAGCCCAACCCTATGAGCCCACAGCAGCACATGCTCCCAAATCAGGC
ACAGTCCCCACACCTACAAGGTCAACAAATCCCTAATTCTCTCTCCAATCA
AGTGCGCTCTCCCCAGCCTGTCCCTTCTCCTCGACCACAGTCTCAGCCCCC
CCATTCCAGCCCATCTCCGAGGATGCAGCCTCAGCCTTCTCCACACCATGT
TTCTCCACAGACCAGTTCCCCACATCCTGGACTGGTAGCTGCCCAGGCTGC
CAACCCTATGGAACAAGGGCATTTTGCCAGCCCAGACCAGAATTCAATGC
TTTCACAGCTCGCTAGCAATCCTGGCATGGCAAACCTCCACGGGGCAAGT
GCCACGGACCTGGGACTGAGCAGCGATAATGCAGACTTGAATTCAAACCT
CTCACAGAGTACACTAGACATACACTAGCCCATATGGTGAGCGTGGACTT
TCCTGAAATGATGGCAGAGATCATCTCTGTGCAAGTGCCCAAGATCCTTTC
TGGGAAAGTCAAGCCCATCTATTTCCACACACAGgactacaaagaccatgacggtgattat
aaagatcatgacatcgattacaaggatgacgatgacaagTGAGCTAGC
