# Supplementary material for: The Activation of p300 Enhances the Sensitivity of Pituitary Adenomas to Dopamine Agonist Treatment by Regulating the Transcription of DRD2
Source: Int J Mol Sci. 2024 Nov 21;25(23):12483. doi: 10.3390/ijms252312483 (PMC11641041; doi:10.3390/ijms252312483)
Supplement: Supplementary file 1 [file ijms-25-12483-s001.zip › ijms-3289906-supplementary Table S3.pdf]

Primers used in CHIP-qPCR and RT-qPCR.

	Gene Name	Forward Primer	Reverse Primer
CHIP-qPCR	Rat DRD2	5'-TCCGTCACCCAGAGCATC-3'	5'-TGGTTGAGGAGACAAGAAGC-3'
	Mouse DRD2	5'-CATCAATCACCCAGAGCATC-3'	5'-AGAAGCAGGAGCCAGCC-3'
RT-qPCR	Human p300	5′-AGCCAAGCGGCCTAAACTC-3	5′-TCACCACCATTGGTTAGTCCC-3
	Rat p300	5′-CTCCCGATCCTGCTGCTTTAAA-3′	5′-TGTCCCATGTTAGGTGCAGTATT-3′
	Mouse p300	5′-CTTCCTCACTGTCGTACCATGAA-3	5′-AGAGGAAGACACACAGGACAATC-3
	Rat DRD2	5′-GGTAATGCCGTGGGTTGTCT-3	5′-TTGTTGAGTCCGAAGAGCAGT-3
	Mouse DRD2	5′-GTCCTGTCCTTCACCATCTCTTG-3	5′-TAGAACGAGACGATGGAGGAGTA-3
	Human GAPDH	5′-TCACCACCATTGGTTAGTCCC-3	5′-GGCTGTTGTCATACTTCTCATGG-3
	Rat GAPDH	5′-GCATCTTCTTGTGCAGTGCC-3′	5′-GATGGTGATGGGTTTCCCGT-3′
	Mouse GAPDH	5′-GCCTCCTCCAATTCAACCCT-3′	5′-CTCGTGGTTCACACCCATCA-3′
