# Supplementary figures and images for: The Activation of p300 Enhances the Sensitivity of Pituitary Adenomas to Dopamine Agonist Treatment by Regulating the Transcription of DRD2
Source: Int J Mol Sci. 2024 Nov 21;25(23):12483. doi: 10.3390/ijms252312483 (PMC11641041; doi:10.3390/ijms252312483)

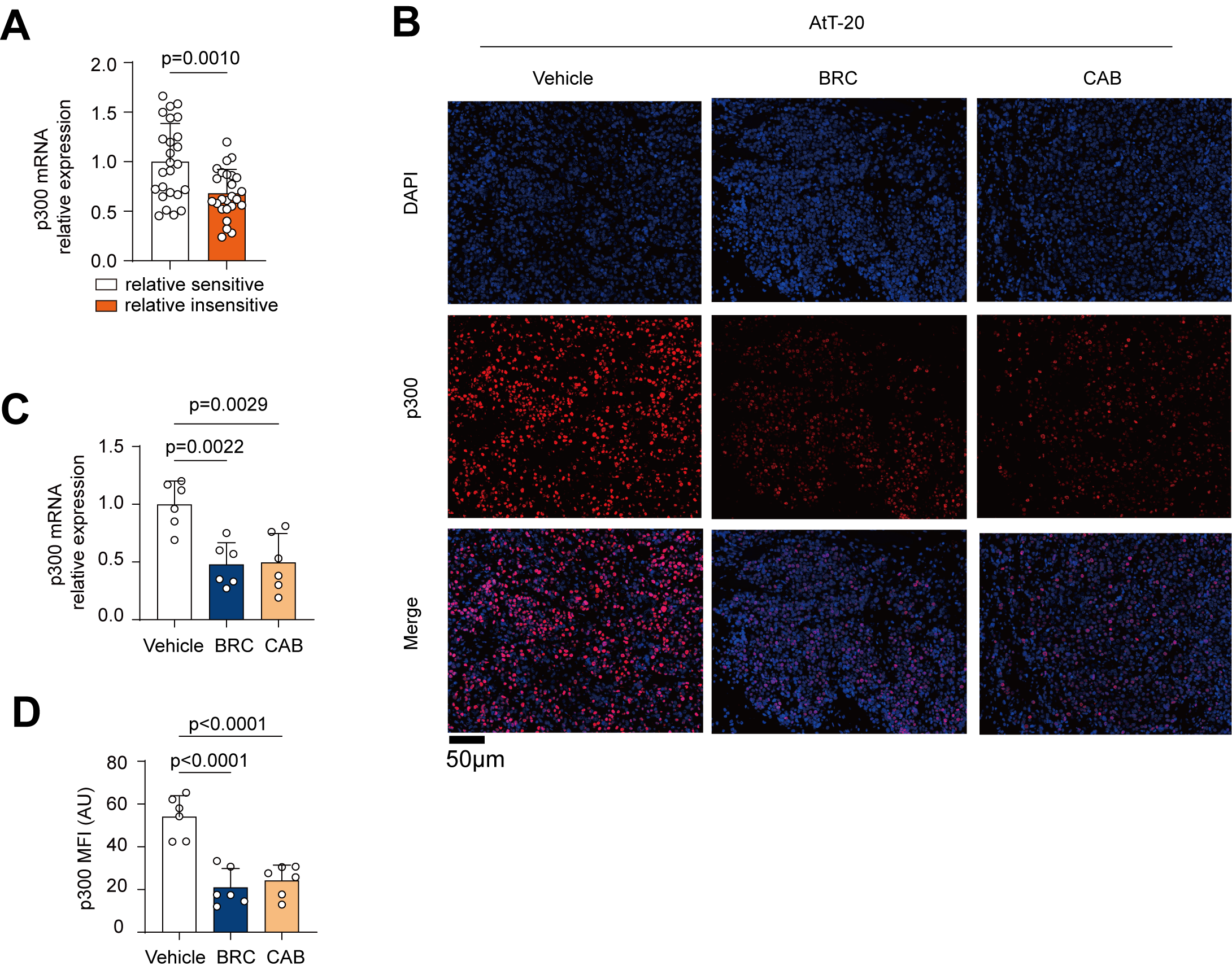

Supplement: Supplementary file 1 [file ijms-25-12483-s001.zip › ijms-3289906-Supplementary Figure S1.tif]

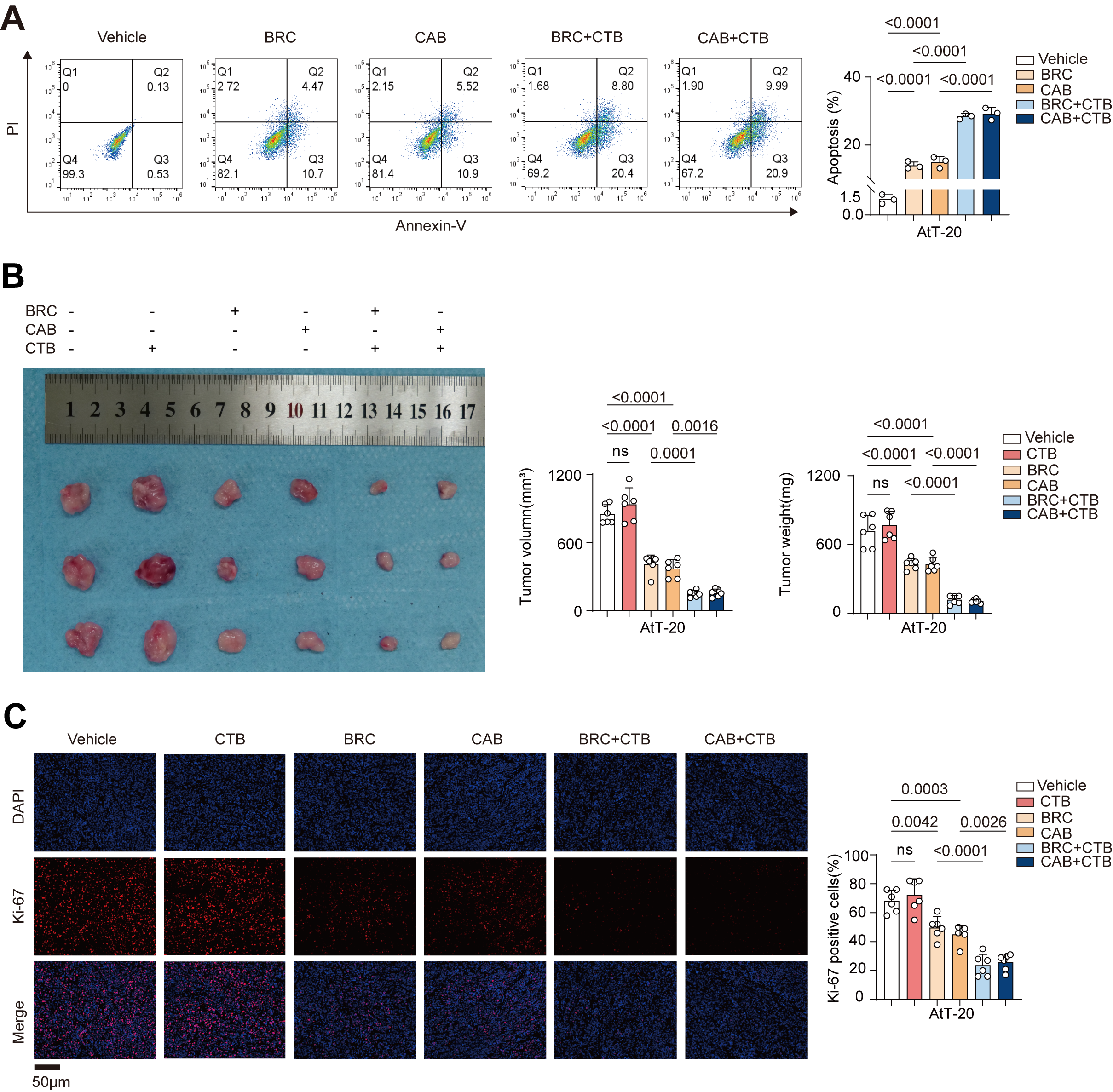

Supplement: Supplementary file 1 [file ijms-25-12483-s001.zip › ijms-3289906-Supplementary Figure S2.tif]

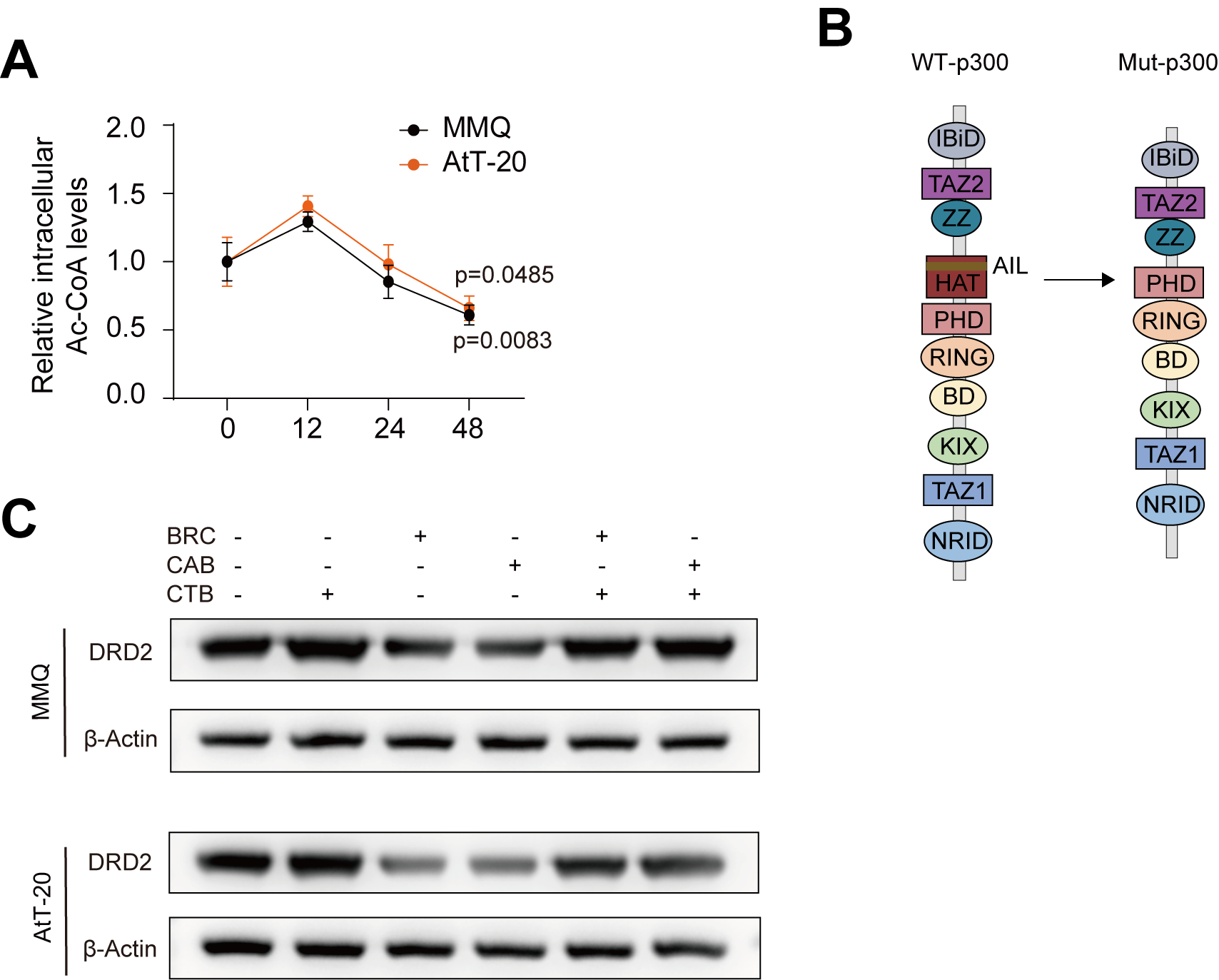

Supplement: Supplementary file 1 [file ijms-25-12483-s001.zip › ijms-3289906-Supplementary Figure S3.tif]

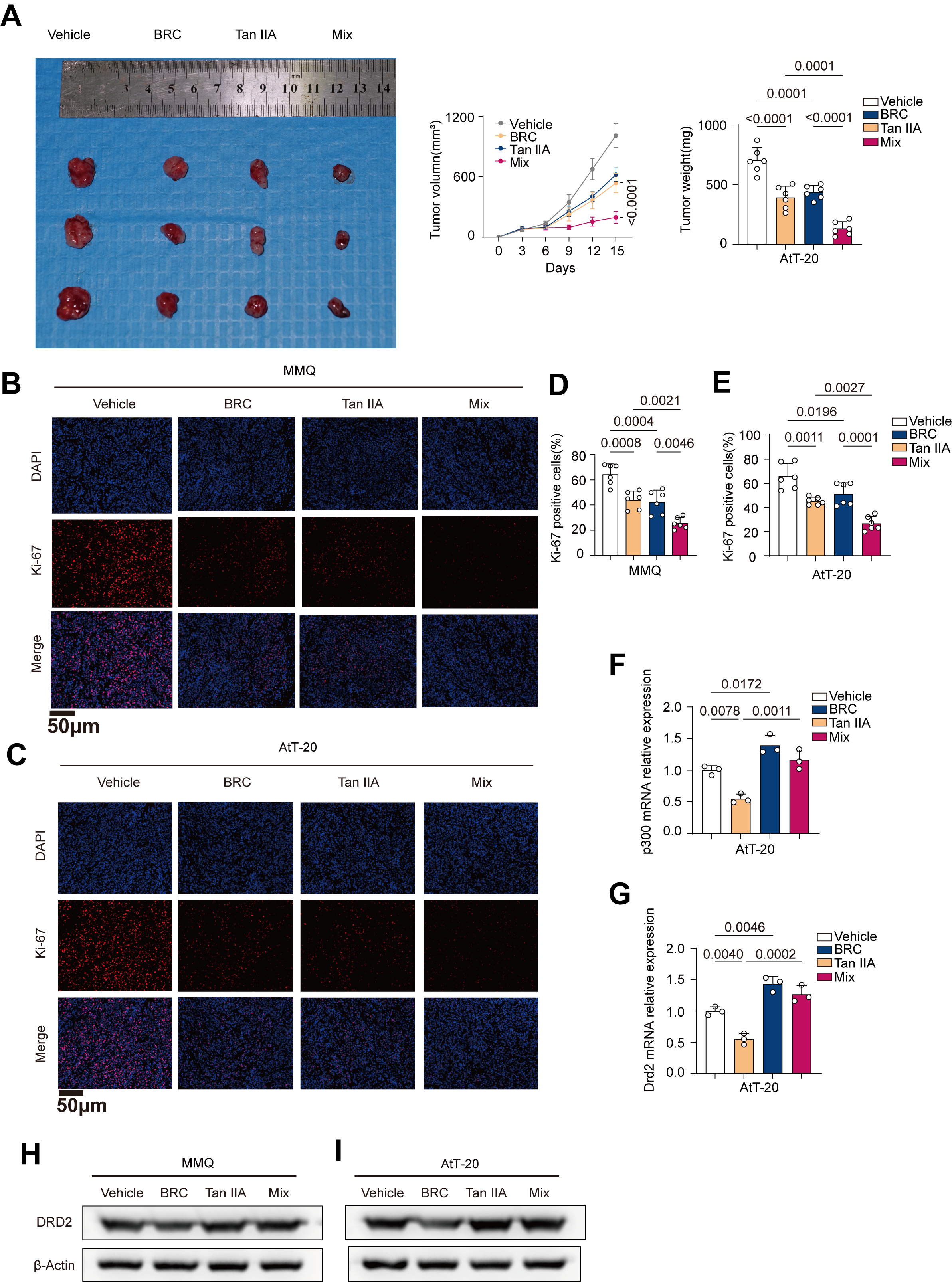

Supplement: Supplementary file 1 [file ijms-25-12483-s001.zip › ijms-3289906-Supplementary Figure S4.tif]
